# Blood pressure changes after stair climbing in busy and quiet outpatient clinic environments among Tanzanian adults: a randomised crossover study

**DOI:** 10.3389/fcvm.2026.1846924

**Published:** 2026-10-05

**Authors:** Issakwisa Habakkuk Mwakyula, Oscar Mahinyila, Thobias Boniphace, Christopher Hariri Mbotwa, Anthony Ambikile Nsojo, Gloria Reginald Mbwile

**Affiliations:** 1 Internal Medicine, Mbeya Zonal Referral Hospital, Mbeya, Tanzania; 2 Internal Medicine, University of Dar es Salaam Mbeya College of Health and Allied Sciences, Mbeya, Tanzania; 3 Social Sciences, University of Dar es Salaam Mbeya College of Health and Allied Sciences, Mbeya, Tanzania; 4 Microbiology and Immunology, Mbeya Zonal Referral Hospital, Mbeya, Tanzania; 5 Microbiology and Immunology, University of Dar es Salaam Mbeya College of Health and Allied Sciences, Mbeya, Tanzania; 6 Pediatric and Child Health, Mbeya Zonal Referral Hospital, Mbeya, Tanzania; 7 Pediatric and Child Health, University of Dar es Salaam Mbeya College of Health and Allied Sciences, Mbeya, Tanzania

**Keywords:** blood pressure measurement, crossover study, hypertension, outpatient care, renal markers, rest duration, Tanzania

## Abstract

**Introduction:**

Blood pressure (BP) measured soon after activity may differ from later seated readings. We examined serial BP changes after stair climbing in busy and quiet outpatient settings, without assuming that a particular waiting period represents complete physiological recovery.

**Methods:**

In this randomized crossover study at Mbeya Zonal Referral Hospital, Tanzania, adults with and without a previous hypertension diagnosis drew folded numbered slips to determine environmental order. Staff recording BP were not blinded. Participants ascended and descended 19 steps before measurements immediately and every 3 min for 30 min in each environment. Mixed-effects models included period, sequence, age, sex and body mass index, with session-specific random slopes and autoregressive residual correlation. Classification was compared with each participant's environment-specific average at 24, 27 and 30 min. Exploratory laboratory analyses accounted for reporting limits and used the available observations for each marker. Clinical sensitivity analyses examined alcohol, diabetes history and medication classes.

**Results:**

The analysis included 443 participants—223 with hypertension and 220 without a previous diagnosis—contributing 9,746 measurement occasions. At 15 min, adjusted busy-minus-quiet systolic BP differences were 2.87 mmHg (95% CI 1.39–4.35) and 4.07 mmHg (2.58–5.56), respectively. Classification disagreement at that time was 10.8% (7.3%–15.5%) in quiet and 7.6% (4.8%–11.9%) in busy settings among participants with hypertension; corresponding values without a previous diagnosis were 2.3% (1.0%–5.2%) and 5.9% (3.5%–9.8%). First-period-only systolic contrasts remained positive but were larger and less precise. Evidence for differences in trajectory shape between diagnostic groups depended on the covariate-adjustment specification. No routine laboratory marker showed a clear adjusted association with the signed SBP difference from the late-session average at 12 or 15 min. Environmental systolic estimates changed little after clinical adjustment within matching samples.

**Conclusions:**

BP changed during seated observation after stair climbing, and early readings disagreed more often with the late-session average. Persistent disagreement and within-session variability do not support 12–15 min as a universally sufficient resting interval. The absence of a pre-exercise resting reference and a no-exercise comparator limits attribution of the decline to recovery from activity.

## 1 Introduction

Hypertension remains a major health challenge in sub-Saharan Africa, where its high prevalence is accompanied by persistent gaps in awareness and treatment (1, 2). Accurate blood pressure (BP) measurement is therefore essential for diagnosis and long-term management, as errors in measurement may affect BP classification and subsequent treatment decisions (3).

Recommendations for office BP measurement include 3–5 min of seated rest, supported positioning and repeated readings (3). In routine outpatient clinics, however, these conditions may be difficult to achieve. Patients may walk between departments, move through queues or climb stairs shortly before reaching the measurement station. In such settings, BP may be recorded soon after activity rather than under resting conditions. How these early readings compare with later seated measurements is an important practical question.

Several studies have examined the influence of rest duration on BP, although their findings differ across populations and measurement protocols. The Best Rest Trial found similar average BP from triplicate measurements after 0, 2 and 5 min of rest overall, but the findings were less consistent among participants with higher systolic BP (4). In untreated hypertension, Sala and colleagues observed a continuing fall in BP during 16 min of seated rest (5). Another study also reported substantial variation in stabilization time; however, their longer waiting-time estimates were extrapolated from a nonlinear model fitted to measurements collected over 11 min (6). These differences limit the application of a single resting interval across outpatient settings.

The measurement environment and recent physical activity also require separate consideration. A randomized crossover trial has examined the effects of noise and public vs. private settings on BP readings (7). Research on post-exercise BP changes provides further context, but the activity protocols and observation periods differ from a brief stair task immediately before triage (8). There is still uncertainty, however, about how BP recorded shortly after routine clinic-related activity compares with later measurements in busy and quiet environments.

The primary aim of this study was therefore to characterize systolic BP during 30 min of seated observation after stair climbing in Tanzanian adults with and without a previous hypertension diagnosis. We also examined time-specific disagreement with late-session BP classification, changes in diastolic BP, pulse rate and pulse pressure, and differences between a busy triage area and a quiet room. As an exploratory aim, we assessed selected serum and urinary markers in relation to the signed SBP difference from the late-session average at 12 and 15 min. The randomized crossover design allowed environmental comparisons within the same participant. Without a pre-activity resting measurement or a no-exercise comparator, the study was intended to describe serial post-activity BP changes, not to establish complete physiological recovery or a minimum resting interval.

## 2 Methods

### 2.1 Study design, participants and recruitment

We conducted a two-period, two-sequence randomized crossover study at Mbeya Zonal Referral Hospital, Tanzania. Data were collected from 3 July 2024 to 11 January 2025. Each participant underwent the activity and measurement protocol in both environments. The within-participant comparison was intended to reduce the influence of stable individual characteristics; differences between diagnostic groups remained observational comparisons (9).

We recruited participants with hypertension from the hypertension clinic. The comparison group comprised adults without a previous hypertension diagnosis who reported being healthy. They did not undergo a separate pre-screening resting BP measurement; we obtained their first study BP immediately after the stair task. We collected demographic, anthropometric, and clinical information before the measurement protocol. We included pre-activity laboratory measurements as exploratory variables. Current medication use, previous glucose testing, diabetes diagnosis and treatment, BP-related lifestyle modification and alcohol use were recorded using a standardized questionnaire.

### 2.2 Eligibility criteria

Participants were eligible if they were aged 18 years or older, had either a previous hypertension diagnosis or no previous diagnosis, and were physically able to ascend and descend stairs. Exclusion criteria were severe symptomatic hypertension, heart failure and pregnancy. Absence of a previous hypertension diagnosis was not treated as confirmation of normotension.

### 2.3 Randomization, allocation concealment and blinding

On each recruitment day, we printed available allocation numbers on individual slips of paper, folded them to hide the numbers, and placed them in a box. Participants who had agreed to take part each drew one folded slip at random. Each allocation number was used only once. Odd numbers were assigned the busy–quiet sequence and even numbers the quiet–busy sequence, with an intended 1:1 allocation ratio. The draw determined the assignment; the number remained hidden until the participant selected a slip.

The number determined environmental order, not diagnostic-group membership. Staff recording BP were not blinded to the assigned order. Staff recorded the starting environment for analysis.

### 2.4 Measurement environments

The busy environment was an open triage area reflecting routine clinical activity, including conversations, movement and queue-related interruptions. The quiet room provided greater privacy and fewer disturbances. Sound levels, crowding and interruption frequency were not measured instrumentally. The comparison therefore involved two pragmatic clinical settings rather than quantified noise exposures.

### 2.5 Stair activity and transition between periods

Before measurements in each environment, participants ascended and descended the same 19-step flight linking the first and second floors of the hypertension clinic. They completed the task at their own pace. The route was standardized, but workload, duration and target heart rate were not; we therefore did not assign a quantified exercise intensity to the task.

After completing the 30 min measurement period in the first environment, participants proceeded directly to the second protocol, including repetition of the stair task. No additional washout interval was scheduled. The first 30 min observation period was not a separate washout after the first environmental exposure.

### 2.6 BP measurement and data recording

BP was measured using an OMRON 10 Series Upper Arm Smart Wireless Blood Pressure Monitor, model BP7465. The exact model is listed in the US Blood Pressure Validated Device Listing (ValidateBP) for a general-adult population, with ANSI/AAMI/ISO 81060-2:2009 validation and a 22–42 cm cuff (10). This listing supports validation under its specified test conditions; it does not validate immediate post-activity measurements in this study.

A study doctor-initiated measurements at the scheduled times. BP and pulse values were captured in the OMRON Connect application on an Android device. Date of birth, anthropometric measurements and clinical information were recorded manually on standardized forms. The app-recorded values were then transferred to the study database and linked to the manual records by participant identification number. This transfer and linkage step was included in the source-document verification described in Section 2.7. Cuff selection followed the manufacturer's mid-arm-circumference guidance. Participants sat with their back supported, legs uncrossed, feet on the floor and the measured arm supported at heart level. They were asked to restrict talking during measurements. Before testing, participants were asked to avoid smoking, caffeine, meals and additional physical activity for at least 30 min; the prescribed stair task was the planned exception to the activity restriction.

Measurements were scheduled immediately after the stair task and every 3 min thereafter to 30 min in each environment. Time zero was an immediate post-activity measurement, not a pre-exercise resting baseline. The post-activity measurement timing was the only planned departure from standard resting preparation. Each scheduled occasion contributed one recorded SBP, DBP and pulse value to the analysis (11).

### 2.7 Outcomes, study size and data completeness

The primary physiological outcome was the systolic blood pressure (SBP) trajectory during 30 min of seated observation after stair climbing. Secondary measurements were diastolic blood pressure (DBP), pulse rate and pulse pressure, calculated as SBP minus DBP. We examined differences between environments and disagreement in elevated/not-elevated BP classification at each scheduled time. We used no cutoff for acceptable disagreement to define a sufficient resting interval. The exploratory laboratory outcome was the signed difference between SBP at 12 or 15 min and the participant's mean SBP at 24, 27 and 30 min within the same environment.

We calculated sample-size using a two-sided significance level of 0.05% and 80% power. For the within-participant environmental comparison, an assumed mean SBP difference of 2 mmHg and a standard deviation of the paired differences of 10 mmHg required 199 participants with complete measurements in both environments. For the comparison between participants with and without a previous hypertension diagnosis, an assumed mean SBP difference of 3 mmHg and a common within-group standard deviation of 10 mmHg yielded a requirement of 176 participants per group, or 352 overall, assuming equal group sizes. Both calculations used the noncentral *t* distribution. These requirements concern individual continuous SBP comparisons rather than the full repeated-measures trajectory or classification-disagreement analyses. Participants, rather than repeated readings, were the independent units in the sample-size calculation.

Recruitment was extended beyond the calculated sample-size requirement to use the remaining EGF ELISA reagents purchased for the exploratory laboratory component and avoid wastage. These kits were intended for research use and could not be used for routine clinical testing. The final analysed cohort comprised 443 participants: 223 with hypertension and 220 without a previous diagnosis. Each participant completed both environmental sessions and contributed all 22 scheduled SBP, DBP and pulse records, yielding 9,746 measurement occasions. Age, sex and BMI were complete, and no imputation was required for the primary BP outcomes or these adjustment variables. Laboratory and clinical questionnaire availability varied by variable; the corresponding denominators and missing-data procedures are reported with those analyses.

Database entries were checked against the collection forms, and confirmed transcription errors were corrected before analysis. All source-verified BP and pulse observations were retained, including extreme values; no observation was excluded solely because of its magnitude or model residual. Figure 1 shows the analysed participants, their assigned environmental sequences and the measurement schedule. Pre-enrolment screening, refusal and exclusion counts are not represented.

**Figure 1:**
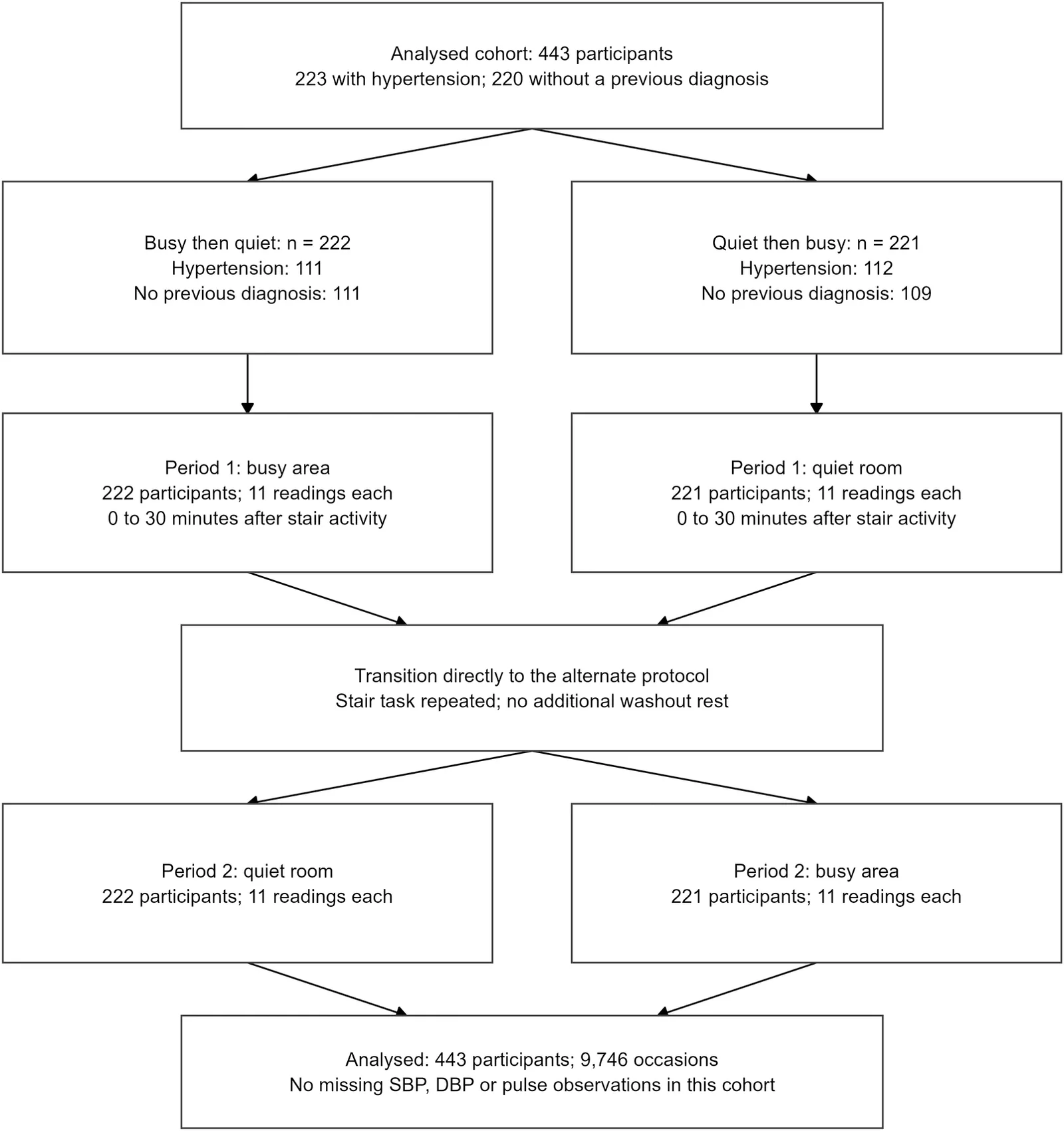
Crossover sequence and measurement schedule among the analysed participants. The analytic cohort comprised 443 participants: 222 followed the busy-then-quiet sequence and 221 the quiet-then-busy sequence. Each period consisted of the stair task followed by 11 scheduled blood pressure (BP) and pulse measurements from 0 to 30 min. Participants proceeded directly to the alternate protocol, including repetition of the stair task, without an additional scheduled washout rest. Counts refer to the analysed cohort; screening, refusal and exclusion counts before inclusion are not represented.

### 2.8 Statistical analyses

Continuous variables are summarized as mean (SD) or median [interquartile range], and categorical variables as counts and percentages. Observed trajectories are presented separately from adjusted estimates. Descriptive mean intervals are pointwise *t*-based 95% confidence intervals.

We fitted linear mixed-effects models by restricted maximum likelihood using nlme. Time was categorical at 0, 3, 6, 9, 12, 15, 18, 21, 24, 27 and 30 min. Fixed effects included time, environment, diagnostic group, their two- and three-way interactions, age represented by a natural cubic spline with three degrees of freedom, sex, BMI, period and sequence. Period identified the first or second observation session; sequence identified busy–quiet or quiet–busy order.

For each outcome, four covariance specifications were fitted to identical observations with the same fixed-effects formula: a participant random intercept; participant and session random intercepts; those intercepts with within-session first-order autoregressive [AR(1)] residual correlation; and a participant random intercept together with session random intercepts and time slopes, plus within-session AR(1) correlation. Session intercept–slope covariance was diagonal, with time scaled by 30 min for the random slope. Reporting-model selection considered AIC, BIC, variance estimates and residual diagnostics. AIC/BIC comparisons were confined to covariance candidates with the same fixed effects and analysis sample.

Estimated marginal means and contrasts used common participant-level covariate averages and proportional weighting of nuisance-factor levels. We report busy-minus-quiet differences and diagnostic-group contrasts with pointwise 95% confidence intervals. Nominal *p*-values and Holm-adjusted *p*-values are provided for the 22 environmental contrasts within each outcome model; the 22 diagnostic-group contrasts constitute a separate family. Between-group estimates are not interpreted as randomized effects of hypertension status.

During revision, we added SBP sensitivity analyses allowing time-by-period variation, restricting analysis to the first period, allowing age-spline terms, sex and BMI to interact with time, and restricting the latter model to the overlapping age range of 25–79 years. We also fitted a separate ordinary least-squares model with participant-clustered covariance. First-period models did not include period or sequence because period was constant and sequence coincided with environment. Period and time-by-period terms were tested separately using their respective denominator degrees of freedom. A supplementary large-sample Wald chi-square test jointly assessed all period-related terms. We did not use a carryover pre-test to decide whether to retain second-period observations.

Antihypertensive medications were recorded as free text and parsed into pharmacological classes. Fixed-dose combinations were counted as exposure to both component classes, and generic ingredients were used for reporting. Uncertain diabetes responses were distinguished from negative responses. Further sensitivity analyses added alcohol-by-time terms to the SBP, DBP and pulse-rate models. Diabetes-by-time and alcohol-by-time terms were added within participants with a definite Yes/No diabetes response. Reference and additionally adjusted models were fitted to the same participants.

Hypertension-only sensitivity analyses examined pulse rate by beta-blocker identification and environmental SBP differences after beta-blocker and diuretic adjustment. Literal generic names and author-confirmed combinations were used first; proposed spelling mappings were examined separately. Missing or uninterpretable lists were not treated as non-use. Models retained period, sequence and the selected covariance structure. Specifications, sample sizes and contrasts are provided in Supplementary Tables S19–S22 and the supplementary clinical methods.

The internal late-session reference was each participant's mean SBP and mean DBP at 24, 27 and 30 min within the same environment. The previously specified stability definition required every consecutive SBP change across 21, 24, 27 and 30 min to be strictly below 5 mmHg. Elevated BP was defined as SBP ≥140 mmHg or DBP ≥90 mmHg. Disagreement meant a different elevated/not-elevated classification at a scheduled occasion from that based on the late-session average. The reference was not an independently measured resting BP or a diagnostic gold standard.

We calculated pointwise Wilson 95% intervals for disagreement proportions. Paired environmental comparisons used exact McNemar tests and percentile-bootstrap intervals for the busy-minus-quiet difference, using 10,000 participant-pair resamples. Holm adjustment covered the 22 group-by-time paired tests. The bootstrap intervals and exact tests use different inferential procedures and are reported separately. As sensitivity analyses, we averaged the recorded values over the 9/12-, 12/15- and 9/12/15-minute windows and repeated the late comparisons after excluding the index reading from its own reference. These temporal averages are not contemporaneous replicate measurements.

We examined normalized residuals, Q–Q plots and residual-pair correlations at exact lags within each participant/environment session. Across-session residual pairs were not combined. Residual-tail departures were retained in the diagnostic figures and considered when interpreting model-based inference. All tests were two-sided. Analyses were run in R 4.6.0 using nlme, emmeans, sandwich and the other packages recorded in the accompanying analysis code and session information.

### 2.9 Laboratory measurements and exploratory analyses

Blood and urine specimens were collected before the first stair task and before serial BP measurements. Serum creatinine and urinary creatinine, sodium, potassium and albumin were measured using a COBAS INTEGRA 400 plus analyser (Roche Diagnostics). Urinary epidermal growth factor (EGF) was measured using the Human EGF Sandwich ELISA Kit, KE00138 (Proteintech). Urine specimens for EGF were tested without predilution. The manufacturer's stated standard-curve range was 125–8,000 pg/mL (12).

Urine sodium entries of 19 represented results below 20 mmol/L and were analysed as left-censored observations. Quantitative EGF analyses were conditional on the kit's stated upper boundary of 8,000 pg/mL. Seven other EGF values outside the kit range were excluded from the first quantitative scenario and treated as censored in a second scenario. The corresponding bounds were retained for sodium/potassium and EGF/creatinine ratios. Reporting-limit codes were not treated as exact concentrations.

Laboratory models used the available observations for each marker. Diagnostic-group comparisons used lognormal regression with censored outcomes where applicable, adjusted for an age spline with three degrees of freedom, sex and BMI. Associations with the signed SBP difference at 12 and 15 min were estimated in separate joint models for log marker and the two environmental BP outcomes, integrating over censored predictor values. The BP models included diagnostic group, age spline, sex, BMI, sequence, environment, environment-by-group and period. A common marker coefficient was estimated for the two environments and reported in mmHg per doubling. Sodium/potassium and EGF/creatinine models also included log potassium and log urine creatinine, respectively.

Pointwise sandwich 95% confidence intervals were calculated. Sensitivity analyses used quantified-only participant-clustered linear models, additional serum-creatinine adjustment for urinary markers and the alternative EGF range scenario. For routine-marker Benjamini–Hochberg adjustment, the original family sizes of seven group comparisons and 14 marker–time comparisons were retained. Conditional EGF tests were adjusted separately within each scenario. These revision-stage analyses remained exploratory. The likelihood specification, covariates, missingness and complete results are described in the supplementary laboratory methods and Supplementary Tables S13–S18.

### 2.10 Ethics statement

The study was approved by the Mbeya Medical Research and Ethics Committee (reference number SZEC-2439/RA/V.1/92a). Written informed consent was obtained from all the participants, and their confidentiality and rights were protected throughout the study.

## 3 Results

### 3.1 Participants and recorded sequences

The final analysis included 443 participants: 223 with hypertension and 220 without a previous diagnosis. Of these, 222 followed the busy–quiet sequence and 221 the quiet–busy sequence (Figure 1). The respective sequence counts were 111/112 among participants with hypertension and 111/109 among those without a previous diagnosis. All analysed participants contributed 22 measurement occasions, giving 9,746 complete occasions.

Participants with hypertension were older and had a higher mean BMI. Mean ages were 63.5 (SD 9.7) and 39.2 (13.5) years, and mean BMI values were 31.4 (5.7) and 27.2 (6.1) kg/m^2^, respectively. Women comprised 161/223 (72.2%) and 158/220 (71.8%). Table 1 gives the main characteristics; Supplementary Table S1 provides the additional anthropometric measures.

**Table 1:** Participant characteristics.

Characteristic	Overall	Hypertension	No previous diagnosis
Participants, *n*	443	223	220
Female, *n* (%)	319 (72.0%)	161 (72.2%)	158 (71.8%)
Age (years)	51.4 (16.9)	63.5 (9.7)	39.2 (13.5)
Weight (kg)	76.2 (16.4)	81.7 (15.3)	70.7 (15.7)
Height (cm)	161.5 (7.7)	161.4 (7.5)	161.5 (8.0)
Body mass index (kg/m^2^)	29.3 (6.3)	31.4 (5.7)	27.2 (6.1)
Waist circumference (cm)	95.0 (15.1)	102.2 (13.6)	87.6 (12.8)
Reported BP medication	222 (50.1%)	222 (99.6%)	0 (0.0%)
Previous diabetes diagnosis: yes	46 (10.4%)	44 (19.7%)	2 (0.9%)
Previous diabetes diagnosis: no	246 (55.5%)	156 (70.0%)	90 (40.9%)
Previous diabetes diagnosis: uncertain	149 (33.6%)	22 (9.9%)	127 (57.7%)
Previous diabetes diagnosis: missing	2 (0.5%)	1 (0.4%)	1 (0.5%)
Reported alcohol use	102 (23.0%)	50 (22.4%)	52 (23.6%)

Values are mean (SD) or *n* (%). Percentages use the full group denominator. Hypertension denotes a previous diagnosis; the comparison group was not independently confirmed normotensive. Diagnostic groups were not randomized. Diabetes history was self-reported; uncertain and missing responses are distinct from No. Medication and alcohol responses do not establish dose, frequency or adherence.

BP medication was reported by 222/223 (99.6%) participants with hypertension and none without a previous diagnosis. A previous diabetes diagnosis was reported by 44/223 (19.7%) and 2/220 (0.9%), respectively; 22 and 127 were uncertain, with one missing response in each group. Alcohol use was reported by 50/223 (22.4%) and 52/220 (23.6%) (Table 1). Full questionnaire distributions and medication-class counts are presented in Supplementary Tables S19, S20.

### 3.2 BP trajectories and adjusted environmental comparisons

Observed mean SBP fell most during the early part of each seated session (Figure 2). In the quiet room, it changed from 148.9 (SD 23.1) mmHg immediately after activity to 128.8 (18.8) mmHg at 15 min among participants with hypertension, and from 127.8 (20.0) to 110.8 (16.2) mmHg among those without a previous diagnosis. These observed means are not the covariate-adjusted estimates.

**Figure 2:**
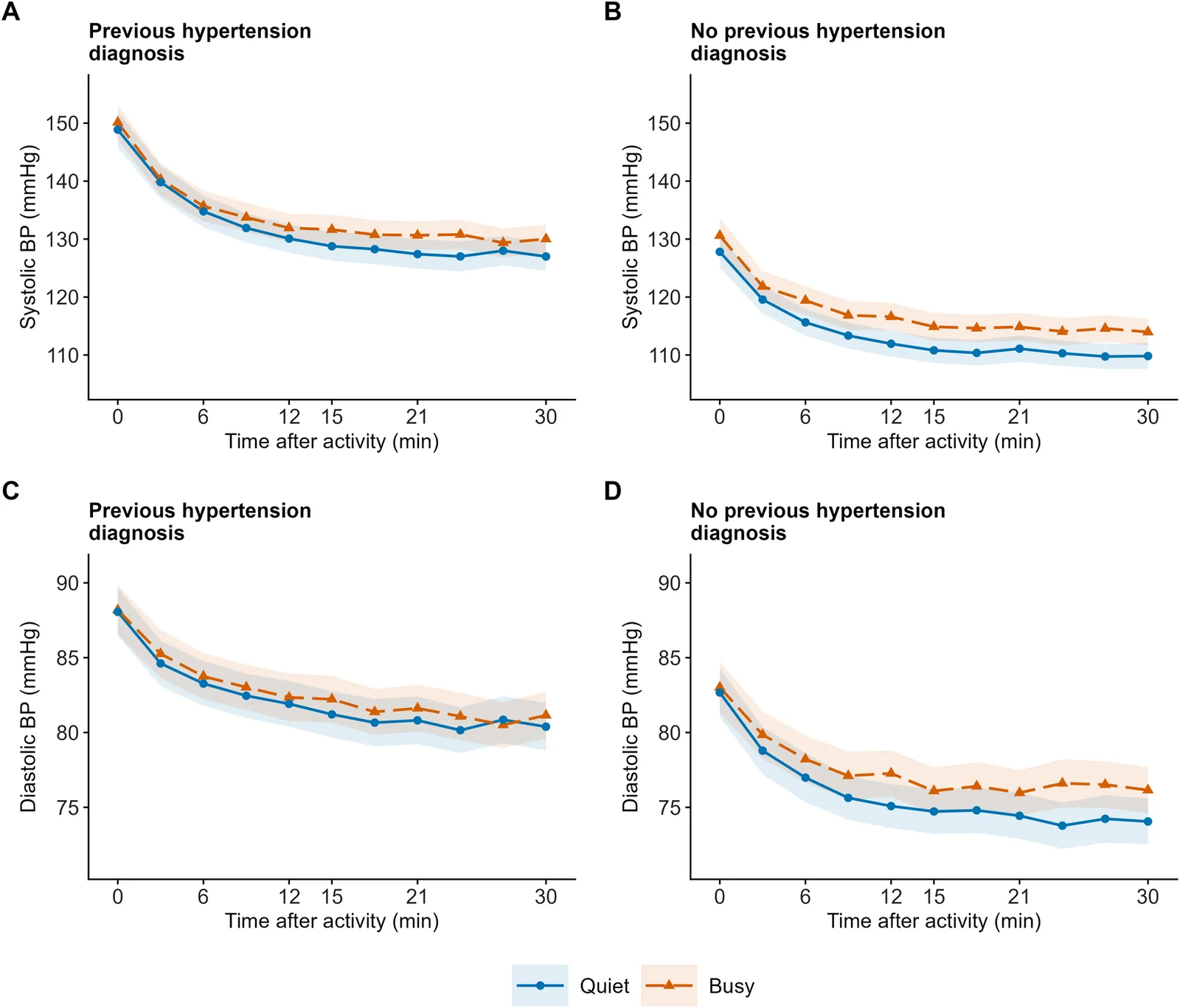
Observed systolic and diastolic BP during seated observation after stair activity. Panels **(A)** and **(B)** show systolic BP (SBP) in participants with and without a previous hypertension diagnosis, respectively; panels **(C)** and **(D)** show the corresponding diastolic BP (DBP). Blue circles and solid lines indicate the quiet room; orange triangles and dashed lines indicate the busy area. Points are unadjusted means, and ribbons are pointwise *t*-based 95% confidence intervals. Each environment/time-point summary includes 223 participants with a previous diagnosis and 220 without a previous diagnosis. Time 0 is the immediate post-activity measurement, not a pre-exercise resting baseline. Lines connect scheduled observations rather than fitted recovery curves.

The model with participant intercepts, session intercepts and time slopes, and within-session AR(1) residual correlation had the lowest AIC and BIC among the four covariance candidates for each outcome (Supplementary Table S2). Estimated AR(1) correlations were 0.379 for SBP, 0.249 for DBP and 0.288 for pulse rate and pulse pressure. Adjusted BP trajectories are shown in Supplementary Figure S1; the full adjusted means and contrasts are in Supplementary Tables S3–S5.

At 15 min, the adjusted busy-minus-quiet SBP difference was 2.87 mmHg (95% CI 1.39–4.35) among participants with hypertension and 4.07 mmHg (2.58–5.56) among those without a previous diagnosis. Corresponding DBP differences were 1.01 mmHg (−0.03 to 2.06) and 1.37 mmHg (0.32–2.42) (Table 2). These are pointwise intervals. The DBP contrast in the latter group had nominal *p* = 0.011 but Holm-adjusted *p* = 0.158. At 15 min in the quiet room, the adjusted SBP difference between diagnostic groups was 5.89 mmHg (0.76–11.01; nominal *p* = 0.024; Holm-adjusted *p* = 0.342).

**Table 2:** Adjusted environmental BP contrasts at selected times.

Group	Time (min)	SBP difference (95% CI)	DBP difference (95% CI)
Previous hypertension diagnosis	0	1.26 (−0.10 to 2.61)	0.12 (−0.84 to 1.09)
Previous hypertension diagnosis	6	0.91 (−0.47 to 2.28)	0.49 (−0.49 to 1.46)
Previous hypertension diagnosis	12	1.83 (0.40 to 3.27)	0.43 (−0.58 to 1.45)
Previous hypertension diagnosis	15	2.87 (1.39 to 4.35)	1.01 (−0.03 to 2.06)
Previous hypertension diagnosis	30	3.02 (1.23 to 4.82)	0.76 (−0.48 to 1.99)
No previous hypertension diagnosis	0	2.80 (1.43 to 4.16)	0.34 (−0.63 to 1.32)
No previous hypertension diagnosis	6	3.78 (2.40 to 5.17)	1.23 (0.25 to 2.22)
No previous hypertension diagnosis	12	4.66 (3.21 to 6.10)	2.18 (1.16 to 3.20)
No previous hypertension diagnosis	15	4.07 (2.58 to 5.56)	1.37 (0.32 to 2.42)
No previous hypertension diagnosis	30	4.15 (2.34 to 5.96)	2.09 (0.85 to 3.34)

Busy minus quiet; positive values indicate higher BP in the busy area. Units: mmHg. Reporting models: categorical time, environment, diagnostic group and all their interactions; age spline (3 df), sex, BMI, period and sequence; participant intercept, session intercept and slope with diagonal covariance, and within-session AR(1). Intervals are pointwise 95%, not multiplicity-adjusted. Complete time-specific nominal and Holm-adjusted *p*-values are supplied in Supplementary Table S4.

### 3.3 Period, sequence and sensitivity analyses

In the SBP model allowing time-by-period variation, neither the period main-effect term [*F*_(1, 440)_ = 0.103; *p* = 0.748] nor the time-by-period interaction [*F*_(10, 8,810)_ = 0.571; *p* = 0.839] was statistically significant. In the reporting models, the DBP sequence term had a nominal association [*F*_(1, 435)_ = 5.234; *p* = 0.023], whereas the SBP sequence test had *p* = 0.065 (Supplementary Table S6). These exploratory tests do not establish the presence or absence of carryover.

First-period-only SBP differences at 15 min were positive but larger and less precise than the full crossover estimates: 7.22 mmHg (95% CI 2.46–11.98) among participants with hypertension and 5.83 mmHg (1.05–10.60) among those without a previous diagnosis. The separate participant-clustered linear model gave corresponding full-data estimates of 2.87 mmHg (1.67–4.07) and 4.07 mmHg (2.92–5.22) (Supplementary Figure S4; Supplementary Table S7).

Evidence for differences in SBP trajectory shape between diagnostic groups depended on the adjustment specification. The joint group-trajectory test had *p* < 0.001 in the reporting model, *p* = 0.076 when age, sex and BMI associations were allowed to vary with time, and *p* = 0.120 in the corresponding age-overlap analysis of 400 participants. Joint environmental-trajectory tests had *p* = 0.0036 and *p* = 0.0081 in these latter two analyses (Supplementary Table S8).

As shown in Supplementary Table S21, alcohol adjustment left the 15 min environmental SBP estimates unchanged at the reported precision. Among the 292 participants with definite diabetes responses, the corresponding differences were 2.68 mmHg (95% CI 1.13–4.24) with hypertension and 4.25 mmHg (1.95–6.55) without a previous diagnosis after diabetes and alcohol adjustment; matched-sample reference estimates were nearly identical (Supplementary Figure S12). Within 176 participants with hypertension and interpretable medication records, additional beta-blocker and diuretic adjustment gave a 15 min difference of 2.50 mmHg (0.76–4.24), compared with 2.50 mmHg (0.75–4.25) in the reference model.

### 3.4 Late-session variation and reference values

The consecutive-reading stability criterion across 21–30 min was met by 64/223 (28.7%) participants with hypertension in the quiet room and 55/223 (24.7%) in the busy area. Corresponding counts without a previous diagnosis were 66/220 (30.0%) and 71/220 (32.3%). The late-session average was elevated in 21/220 (9.5%) comparison participants in the quiet room and 30/220 (13.6%) in the busy area (Supplementary Table S10a). These measurements neither confirm nor exclude previously unrecognized hypertension.

### 3.5 Classification disagreement and temporal averaging

Disagreement with late-session classification was lower at 15 min than immediately after activity but did not decrease at every successive measurement (Figure 3; Table 3). Among participants with hypertension, quiet-room disagreement changed from 90/223 (40.4%) immediately after activity to 24/223 (10.8%; 95% CI 7.3–15.5) at 15 min. Busy-area disagreement changed from 84/223 (37.7%) to 17/223 (7.6%; 4.8%–11.9%). Without a previous diagnosis, the corresponding changes were 53/220 (24.1%) to 5/220 (2.3%; 1.0%–5.2%) in the quiet room, and 57/220 (25.9%) to 13/220 (5.9%; 3.5%–9.8%) in the busy area.

**Figure 3:**
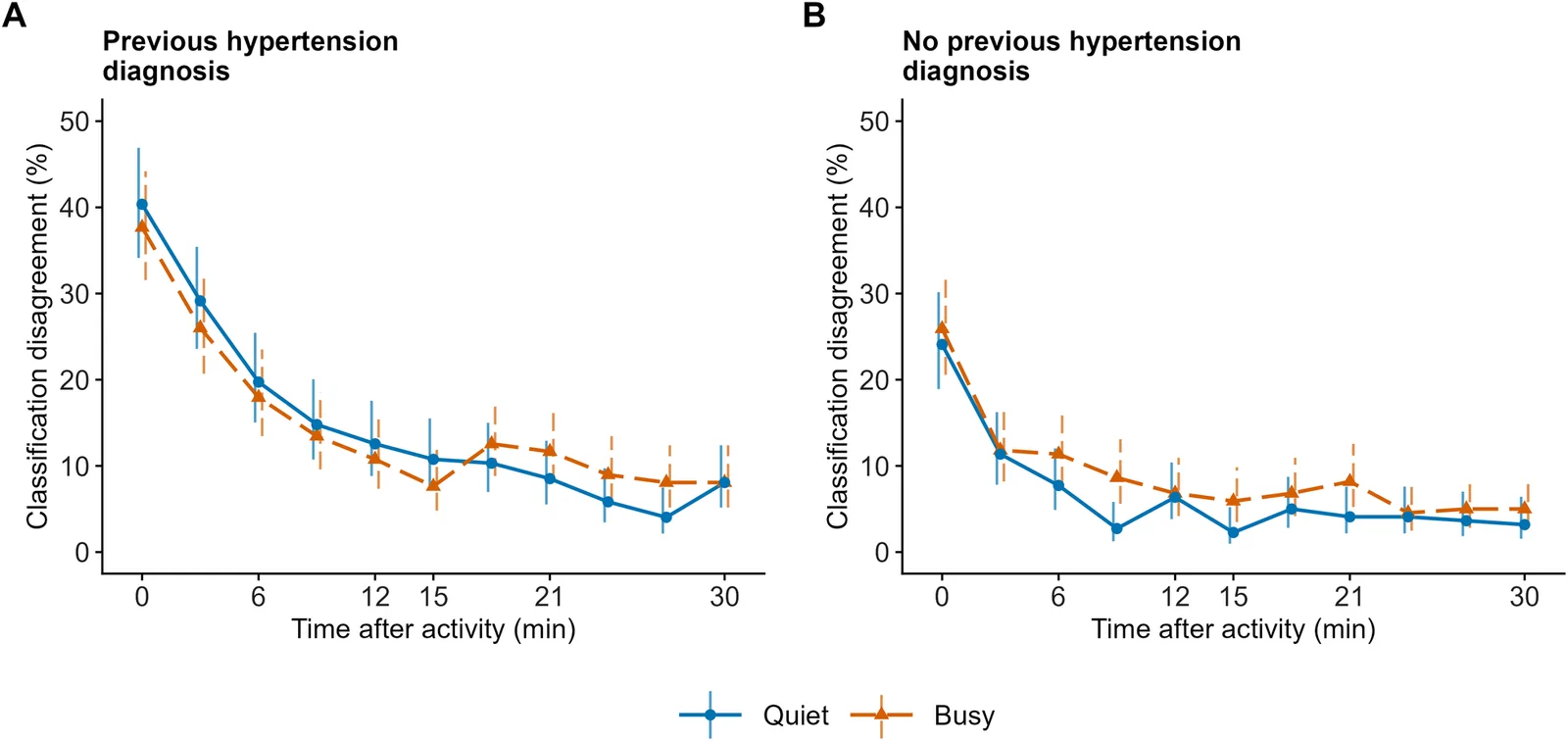
Classification disagreement relative to the internal late-session average. Panels **(A)** and **(B)** represent participants with and without a previous hypertension diagnosis, respectively. Blue circles/solid lines indicate quiet; orange triangles/dashed lines indicate busy. Points are disagreement proportions, with pointwise Wilson 95% confidence intervals displaced slightly horizontally for visibility. Elevated BP is SBP ≥ 140 mmHg or DBP ≥ 90 mmHg. The internal reference uses each participant's mean SBP and mean DBP at 24, 27 and 30 min within the same environment, rather than an independently established resting BP or diagnosis. Each of those three late index readings contributes to its own reference; leave-one-out results are in Supplementary Table S10c. No acceptable-disagreement threshold or sufficient resting interval is defined.

**Table 3:** Disagreement with the late-session BP classification.

Group	Time (min)	Quiet: *n*/*N*; % (95% CI)	Busy: *n*/*N*; % (95% CI)
Previous hypertension diagnosis	0	90/223; 40.4% (34.1–46.9)	84/223; 37.7% (31.6–44.2)
Previous hypertension diagnosis	6	44/223; 19.7% (15.0–25.4)	40/223; 17.9% (13.5–23.5)
Previous hypertension diagnosis	12	28/223; 12.6% (8.8–17.5)	24/223; 10.8% (7.3–15.5)
Previous hypertension diagnosis	15	24/223; 10.8% (7.3–15.5)	17/223; 7.6% (4.8–11.9)
Previous hypertension diagnosis	30	18/223; 8.1% (5.2–12.4)	18/223; 8.1% (5.2–12.4)
No previous hypertension diagnosis	0	53/220; 24.1% (18.9–30.2)	57/220; 25.9% (20.6–32.1)
No previous hypertension diagnosis	6	17/220; 7.7% (4.9–12.0)	25/220; 11.4% (7.8–16.2)
No previous hypertension diagnosis	12	14/220; 6.4% (3.8–10.4)	15/220; 6.8% (4.2–10.9)
No previous hypertension diagnosis	15	5/220; 2.3% (1.0–5.2)	13/220; 5.9% (3.5–9.8)
No previous hypertension diagnosis	30	7/220; 3.2% (1.5–6.4)	11/220; 5.0% (2.8–8.7)

Pointwise Wilson 95% intervals. Reference: environment-specific mean SBP/DBP at 24, 27 and 30 min. Elevated BP: SBP ≥ 140 or DBP ≥ 90 mmHg. This is disagreement with an internal reference, not diagnostic misclassification against an independent gold standard. The 30 min index contributes to its reference; Supplementary Table S10c gives leave-one-out results. Supplementary Table S9b gives paired comparisons. No acceptable-recovery cutoff is assumed.

At 15 min, the paired busy-minus-quiet disagreement difference was −3.14 percentage points (bootstrap 95% CI −7.62 to 1.35; exact McNemar *p* = 0.248) among participants with hypertension. Without a previous diagnosis, it was 3.64 percentage points (0.45–7.27; exact McNemar *p* = 0.077). The latter interval and exact test were calculated by different methods. None of the 22 paired exact tests remained below 0.05 after Holm adjustment (Supplementary Tables S9a,b).

Mean absolute SBP differences from the late-session average remained 5.35 mmHg in quiet and 5.53 mmHg in busy settings at 15 min among participants with hypertension; corresponding values without a previous diagnosis were 4.42 and 4.51 mmHg (Supplementary Figure S2). Averaging recorded BP at 12 and 15 min gave disagreement proportions of 9.4% in quiet and 8.5% in busy settings among participants with hypertension, and 4.5% in each environment without a previous diagnosis. Full temporal-window and leave-one-out results are presented in Supplementary Tables S10b,c.

### 3.6 Secondary measurements and model diagnostics

Observed pulse-rate and pulse-pressure trajectories are presented in Supplementary Figure S3, with their adjusted means and contrasts in Supplementary Tables S3–S5. The principal models did not include medication classes; additional pulse-rate analyses are reported below. Residual plots showed appreciable tail departures, particularly for pulse rate, and residual-pair correlations were not uniformly zero at short lags (Supplementary Figures S5–S8; Supplementary Table S12). The main and sensitivity estimates are therefore reported together rather than treating the selected covariance structure as proof that every model assumption was satisfied.

Among 182 participants with hypertension and interpretable beta-blocker records, beta-blocker identification was associated with a lower modelled pulse rate at 15 min (difference −6.23 beats/min; pointwise 95% CI −10.29 to −2.17; nominal *p* = 0.003; Holm-adjusted *p* = 0.062). The joint time-by-beta-blocker test was not statistically significant [Wald *χ*^2^_(10)_ = 8.56; *p* = 0.574]. Including proposed medication spellings increased the sample to 199 and gave a difference of −6.37 beats/min (−10.22 to −2.53), with a joint trajectory *p*-value of 0.745. The common beta-blocker contrast was constrained to be the same in both environments (Supplementary Table S22).

### 3.7 Exploratory laboratory findings

Serum creatinine was available for 430 participants, routine urine chemistry and uACR for 416, and EGF for 428. Sodium was below 20 mmol/L in 24 participants. The EGF data included 64 above-range codes and seven other out-of-calibration values (Supplementary Table S13; Supplementary Figure S9). After adjustment for age, sex and BMI, all five routine diagnostic-group comparison intervals included a ratio of 1, and all BH *q*-values were at least 0.492. The adjusted ratio was 1.01 (95% CI 0.93–1.09) for serum creatinine and 0.94 (0.78–1.13) for urine sodium (Supplementary Table S15).

None of the routine-marker associations with the signed SBP difference at 12 or 15 min had a 95% confidence interval excluding zero; all routine-marker BH *q*-values were at least 0.758 (Supplementary Table S16; Supplementary Figure S10). For uACR at 15 min, the estimate was 0.24 mmHg per doubling (95% CI −0.02 to 0.51). Quantified-only analyses and analyses with additional serum-creatinine adjustment also produced intervals crossing zero, although some point estimates changed direction. Neither conditional EGF scenario provided clear evidence of a group difference or a marker–BP association (Supplementary Tables S15–S17; Supplementary Figure S11).

## 4 Discussion

BP measured immediately after stair climbing differed from later seated measurements in both outpatient environments. The largest average decline occurred during the early part of each session, and systolic values were generally higher in the busy area. Classification disagreement was lower after 12–15 min, but individual variation persisted. These findings do not establish a universally sufficient resting interval.

The distinction between an observed decline and physiological recovery is important. We did not measure BP before activity or include a no-exercise condition. Recovery from exertion may have contributed, but acclimatization to the setting, repeated-measurement effects and regression to the mean remain plausible explanations. Post-exercise hypotension after more sustained exercise is also not the same outcome as return toward a pre-activity value (8). Our data cannot separate these mechanisms.

Previous rest-duration studies help place these findings in context. The Best Rest Trial used triplicate measurements, randomized rest intervals and a noninferiority framework; similar overall averages after shorter rest do not directly establish the appropriate waiting period after stair climbing (4). Sala and colleagues documented a continuing seated BP fall in untreated hypertension (5). Mahe and colleagues modelled variation in stabilization time, but their proposed longer intervals extended beyond the observed measurement period (6). Differences in population, preceding activity, reference definition and measurement averaging therefore limit direct comparisons between studies.

Our environmental comparison reflected routine clinic conditions. Unlike the trial of noise and public settings (7), we did not measure decibels, crowding or interruptions. The estimated differences therefore apply to the two settings as a whole rather than to noise alone. The earlier trial used triplicate measurements after seated rest, whereas our measurements followed a stair task. Whether recent activity modifies an environmental effect remains a hypothesis that this study cannot test directly. The SBP contrasts were similar in the mixed-model and participant-clustered analyses. First-period-only estimates were larger and less precise, and their magnitude should not be described as unchanged. Restricting the analysis to one period also changes the environmental comparison from within-participant to between-participant.

Period and sequence adjustment improved the analysis, but it cannot establish that direct crossover was free of carryover. The absence of an SBP time-by-period association was not an equivalence test. The nominal DBP sequence association may reflect differences between sequence groups, carryover or chance; this design cannot distinguish those explanations reliably. We therefore retained both periods for the principal analysis and reported the first-period sensitivity without using a preliminary significance test to select the dataset (9).

Comparisons between diagnostic groups require particular caution. Participants with hypertension were substantially older and had greater adiposity. Adjustment for age, sex and BMI accounts for these recorded characteristics but does not establish that residual confounding has been removed. The BMI-adjusted estimates describe conditional group differences; they do not establish a causal pathway through adiposity. Limited overlap in the age distributions also makes some adjusted comparisons dependent on model-based extrapolation. We examined this dependence by restricting a sensitivity analysis to the shared age range. Evidence for differences in group trajectories varied with the adjustment specification, particularly when covariate associations were allowed to change over time. Elevated late-session readings among participants without a previous diagnosis also suggest possible unrecognized hypertension (13). They do not establish its prevalence or justify describing the comparison group as normotensive.

The medication and clinical-history analyses provide additional context. Within matching samples, the environmental SBP estimates changed little after the specified adjustments. This does not establish an absence of treatment confounding: almost all participants with hypertension reported BP medication, while none in the comparison group did, so their independent associations could not be separated reliably. Diabetes history was uncertain in 149 participants and missing in two. Medication doses, adherence, treatment duration and timing were not captured sufficiently for further adjustment, and alcohol use was recorded only as yes/no.

Individual variability remained relevant even when group means changed little. At 15 min, disagreement among participants with hypertension was still 10.8% in quiet and 7.6% in busy settings. Only about one quarter to one third met the strict late consecutive-reading stability criterion. The mean of three late readings was a useful internal comparator, but it was not independently validated. Each of the final three observations also contributed to that average; the leave-one-out analyses address this self-inclusion without turning the reference into a gold standard.

The recording and measurement process adds another limitation. Each scheduled time contributed one recorded BP value, whereas the reference averaged three scheduled values. These quantities need not have the same variability. The analytic file does not establish the within-device averaging setting. Averaging neighboring scheduled times tests one aspect of variability, but it is not equivalent to taking replicate readings at a fixed time after activity. Standard preparation, appropriate cuff fit and repeated averaged readings remain relevant to clinical assessment (3).

The laboratory analyses addressed an exploratory question included in the original study. The adjusted estimates did not provide clear evidence of differences between diagnostic groups or associations with the signed SBP difference at 12–15 min. These findings do not establish biological equivalence or exclude associations under other conditions. Missing laboratory results and unmeasured treatment characteristics may still have influenced the estimates. Adjustment for serum creatinine is not equivalent to measuring GFR or accounting for all kidney disease, and spot-urine concentrations do not measure 24 h excretion. The full exploratory findings are therefore reported in the supplement and are not used to recommend a biomarker-based resting interval.

The study's strengths are its within-participant environmental comparison, random allocation by concealed-number draw, and complete repeated measurements over two 30 min sessions. Its limitations include the lack of an independent resting reference and no-exercise comparator, the absence of an additional washout, unquantified environmental conditions, and no blinding of measurement staff. Automated oscillometric measurement does not remove possible differences in timing, interaction or implementation when staff know the order. The supplementary sample-size assessment concerns single SBP contrasts rather than the full repeated-measures model. Pre-enrolment flow counts were not available for this report. All classification analyses used the single 140/90 mmHg threshold, and disagreement proportions are threshold-dependent; a different threshold, such as 130/80 mmHg, would yield different values. Residual-tail departures and the many time-specific comparisons further limit strong claims based on individual *p*-values.

In practice, these results argue against treating a reading taken immediately after movement through the clinic as interchangeable with a later seated value. They do not show that delaying every measurement by the same interval improves diagnosis, treatment decisions or patient outcomes. A subsequent study should obtain an independent resting reference, document the measurement mode, use repeated averaged readings and define acceptable disagreement before recruitment. These steps are needed before recommending a specific post-activity waiting time.

## 5 Conclusion

BP changed during serial seated observation following stair climbing in both outpatient environments. Disagreement with late-session classification was lower after 12–15 min than immediately after activity, but disagreement and within-session variability persisted. These results do not establish a universally sufficient post-activity resting interval. Studies using an independent resting reference, repeated averaged readings and prespecified acceptability criteria are needed before recommending a specific waiting period. The exploratory laboratory findings do not establish a biomarker-based approach to selecting a resting interval.

## Data Availability

The raw data supporting the conclusions of this article will be made available by the authors, without undue reservation.
