# Supplementary material for: Blood pressure changes after stair climbing in busy and quiet outpatient clinic environments among Tanzanian adults: a randomised crossover study
Source: Front Cardiovasc Med. 2026 Oct 5;13:1846924. doi: 10.3389/fcvm.2026.1846924 (PMC13677844; doi:10.3389/fcvm.2026.1846924)
Supplement: Supplementary file 1 [file Datasheet1.docx]

**Figure S1**

Figure S1. Covariate-adjusted BP means and pointwise 95% confidence intervals from the reporting models. Models include categorical time, environment, diagnostic group, all their interactions, age spline (3 degrees of freedom), sex, BMI, period and sequence. Participant random intercepts, session random intercepts and slopes (diagonal covariance), and within-session AR(1) residual correlation are included. Marginal means use common covariate averages and proportional weights for nuisance factor levels; they are not observed group-specific means. Panel ordering follows Figure 2.

**Figure S2**

Figure S2. Mean absolute difference between the time-point SBP and the same participant's environment-specific late average. Ribbons are pointwise t-based 95% confidence intervals. Panel ordering follows Figure 3. This is an internal comparison, not absolute error against true resting BP or an estimate of device error. Self-inclusion affects the final three time points.

**Figure S3**

Figure S3. Secondary observed pulse-rate and pulse-pressure trajectories. A/B: pulse rate; C/D: pulse pressure, in participants with/without a previous hypertension diagnosis. Pulse pressure is SBP minus DBP. Points/ribbons are unadjusted means/pointwise t-based 95% confidence intervals. No medication-adjusted inference is made from these curves.

**Figure S4**

Figure S4. SBP environmental contrasts at 15 minutes across the specified analyses, with pointwise 95% confidence intervals. First-period-only estimates use 443 participants and 4,873 observations; age-overlap estimates use 400 participants and 8,800 observations. The OLS analysis has participant-clustered covariance and is a separate estimator, not robust standard errors for the mixed model. The first-period estimates are larger and less precise; directional agreement does not imply identical effect magnitude.

**Figure S5**

Figure S5. SBP diagnostic plots for the reporting model. A: normalized conditional residuals versus fitted values; B: normal Q-Q plot; C: pooled Pearson correlations of normalized residual pairs at exact lags within participant/environment sessions. Lag zero is omitted. No across-session pairs or significance bands are used. All verified observations are retained. These descriptive diagnostics do not prove covariance adequacy or normality.

**Figure S6**

Figure S6. DBP diagnostic plots for the reporting model. A: normalized conditional residuals versus fitted values; B: normal Q-Q plot; C: pooled Pearson correlations of normalized residual pairs at exact lags within participant/environment sessions. Lag zero is omitted. No across-session pairs or significance bands are used. All verified observations are retained. These descriptive diagnostics do not prove covariance adequacy or normality.

**Figure S7**

Figure S7. PR diagnostic plots for the reporting model. A: normalized conditional residuals versus fitted values; B: normal Q-Q plot; C: pooled Pearson correlations of normalized residual pairs at exact lags within participant/environment sessions. Lag zero is omitted. No across-session pairs or significance bands are used. All verified observations are retained. These descriptive diagnostics do not prove covariance adequacy or normality.

**Figure S8**

Figure S8. PP diagnostic plots for the reporting model. A: normalized conditional residuals versus fitted values; B: normal Q-Q plot; C: pooled Pearson correlations of normalized residual pairs at exact lags within participant/environment sessions. Lag zero is omitted. No across-session pairs or significance bands are used. All verified observations are retained. These descriptive diagnostics do not prove covariance adequacy or normality.

**Figure S9**

Figure S9. Availability and reporting status of the laboratory measurements. Counts distinguish quantified, below-range, above-range, unresolved calibration and missing results. The EGF upper bound used in quantitative sensitivity analyses assumes the study curve agreed with the supplied kit. The seven unresolved calibration entries remain separate here. Ratios are unavailable when either component is unavailable. Quantified labels describe numerical records accepted for analysis, not independent validation of undiluted EGF testing.

**Figure S10**

Figure S10. Adjusted associations of routine laboratory markers with signed SBP difference at 12 minutes (A) and 15 minutes (B). Points and horizontal lines show mmHg per doubling with pointwise participant-score sandwich 95% intervals. The outcome is the difference from the same-participant, same-environment average at 24,27,30 minutes. Censored sodium and sodium/potassium predictors are integrated over their intervals. Models include clinical covariates, environmental sequence and period; Sodium/potassium and EGF/creatinine models also include log potassium and log urine creatinine, respectively; uACR models do not add urine creatinine. These associations are exploratory and do not establish a resting interval. These laboratory models have no additional medication, diabetes or alcohol adjustment.

**Figure S11**

Figure S11. Conditional EGF sensitivity analyses at 12 minutes (A) and 15 minutes (B). The first scenario treats the confirmed 9900 codes as above the kit's 8000-pg/mL standard and leaves seven other out-of-calibration records unresolved. The second applies the kit's 125/8000 boundaries to all out-of-range entries. Neither scenario validates undiluted urine testing. EGF/creatinine models condition on urinary creatinine; they do not use 9900/creatinine as an exact ratio.

**Figure S12**

Figure S12. Clinical sensitivity analyses of the environmental SBP contrast at 15 minutes. Panels A and B show participants with and without a previous hypertension diagnosis. Points and horizontal lines represent busy-minus-quiet estimates and pointwise 95% confidence intervals. Definite diabetes history means a Yes/No response, not that everyone in the subset has diabetes. Reference and additionally adjusted models are compared within matching samples. These are sensitivity analyses, not randomized effects of alcohol or diabetes.
