# Supplementary material for: Blood pressure changes after stair climbing in busy and quiet outpatient clinic environments among Tanzanian adults: a randomised crossover study
Source: Front Cardiovasc Med. 2026 Oct 5;13:1846924. doi: 10.3389/fcvm.2026.1846924 (PMC13677844; doi:10.3389/fcvm.2026.1846924)
Supplement: Supplementary file 2 [file Table1.docx]

**Table S1. Full available participant characteristics.**

| **Characteristic** | **Overall** | **Hypertension** | **No previous diagnosis** |
| --- | --- | --- | --- |
| Participants, n | 443 | 223 | 220 |
| Female, n (%) | 319 (72.0%) | 161 (72.2%) | 158 (71.8%) |
| Age (years) | 51.4 (16.9) | 63.5 (9.7) | 39.2 (13.5) |
| Weight (kg) | 76.2 (16.4) | 81.7 (15.3) | 70.7 (15.7) |
| Height (cm) | 161.5 (7.7) | 161.4 (7.5) | 161.5 (8.0) |
| Body mass index (kg/m²) | 29.3 (6.3) | 31.4 (5.7) | 27.2 (6.1) |
| Waist circumference (cm) | 95.0 (15.1) | 102.2 (13.6) | 87.6 (12.8) |
| Hip circumference (cm) | 107.1 (13.4) | 111.0 (12.8) | 103.1 (12.8) |
| Total body fat (%) | 36.9 (12.7) | 39.3 (11.9) | 34.4 (12.9) |
| Visceral fat (recorded units) | 9.4 (4.5) | 11.7 (4.5) | 7.1 (3.2) |
| Mean (SD) or n (%). Values describe the analytic cohort, not separate randomized diagnostic groups. All denominators are complete for these variables. | | | |

**Table S2. Covariance candidates fitted by REML.**

| **Outcome** | **Covariance** | **Participants** | **Observations** | **AIC** | **BIC** | **Delta AIC** | **AR(1) rho** |
| --- | --- | --- | --- | --- | --- | --- | --- |
| SBP | Participant intercept | 443 | 9746 | 67983.9 | 68364.4 | 2567.3 |  |
| SBP | Participant + session intercepts | 443 | 9746 | 66851.7 | 67239.4 | 1435.1 |  |
| SBP | Intercepts + AR(1) | 443 | 9746 | 65503.6 | 65898.4 | 87.0 | 0.493 |
| SBP | Session slope + AR(1) | 443 | 9746 | 65416.6 | 65818.6 | 0.0 | 0.379 |
| DBP | Participant intercept | 443 | 9746 | 61405.0 | 61785.5 | 2377.7 |  |
| DBP | Participant + session intercepts | 443 | 9746 | 59875.1 | 60262.8 | 847.8 |  |
| DBP | Intercepts + AR(1) | 443 | 9746 | 59151.8 | 59546.7 | 124.5 | 0.345 |
| DBP | Session slope + AR(1) | 443 | 9746 | 59027.3 | 59429.4 | 0.0 | 0.249 |
| PR | Participant intercept | 443 | 9746 | 59195.3 | 59575.8 | 1806.3 |  |
| PR | Participant + session intercepts | 443 | 9746 | 58200.4 | 58588.1 | 811.4 |  |
| PR | Intercepts + AR(1) | 443 | 9746 | 57445.0 | 57839.9 | 56.0 | 0.358 |
| PR | Session slope + AR(1) | 443 | 9746 | 57389.0 | 57791.1 | 0.0 | 0.288 |
| PP | Participant intercept | 443 | 9746 | 67262.7 | 67643.2 | 1484.1 |  |
| PP | Participant + session intercepts | 443 | 9746 | 66472.7 | 66860.4 | 694.0 |  |
| PP | Intercepts + AR(1) | 443 | 9746 | 65810.4 | 66205.3 | 31.8 | 0.337 |
| PP | Session slope + AR(1) | 443 | 9746 | 65778.6 | 66180.7 | 0.0 | 0.288 |
| AIC/BIC comparisons are restricted to one outcome and identical fixed-effects formulas and observations. Do not compare these values against models using different fixed effects or participants. Session-slope models use diagonal intercept/slope covariance. Lowest AIC/BIC is not proof that all model assumptions are satisfied. | | | | | | | |

**Table S3. Full adjusted means from the reporting models.**

| **Outcome** | **Group** | **Environment** | **Time (min)** | **Adjusted mean (95% CI)** |
| --- | --- | --- | --- | --- |
| SBP | No previous hypertension diagnosis | Quiet | 0 | 133.88 (130.81 to 136.95) |
| SBP | No previous hypertension diagnosis | Quiet | 3 | 125.64 (122.57 to 128.71) |
| SBP | No previous hypertension diagnosis | Quiet | 6 | 121.71 (118.63 to 124.78) |
| SBP | No previous hypertension diagnosis | Quiet | 9 | 119.42 (116.35 to 122.50) |
| SBP | No previous hypertension diagnosis | Quiet | 12 | 118.02 (114.93 to 121.11) |
| SBP | No previous hypertension diagnosis | Quiet | 15 | 116.88 (113.79 to 119.98) |
| SBP | No previous hypertension diagnosis | Quiet | 18 | 116.44 (113.33 to 119.55) |
| SBP | No previous hypertension diagnosis | Quiet | 21 | 117.17 (114.05 to 120.29) |
| SBP | No previous hypertension diagnosis | Quiet | 24 | 116.37 (113.23 to 119.52) |
| SBP | No previous hypertension diagnosis | Quiet | 27 | 115.82 (112.66 to 118.98) |
| SBP | No previous hypertension diagnosis | Quiet | 30 | 115.90 (112.72 to 119.08) |
| SBP | Previous hypertension diagnosis | Quiet | 0 | 142.88 (139.84 to 145.92) |
| SBP | Previous hypertension diagnosis | Quiet | 3 | 133.82 (130.78 to 136.86) |
| SBP | Previous hypertension diagnosis | Quiet | 6 | 128.79 (125.75 to 131.84) |
| SBP | Previous hypertension diagnosis | Quiet | 9 | 125.92 (122.87 to 128.97) |
| SBP | Previous hypertension diagnosis | Quiet | 12 | 124.09 (121.03 to 127.15) |
| SBP | Previous hypertension diagnosis | Quiet | 15 | 122.77 (119.71 to 125.84) |
| SBP | Previous hypertension diagnosis | Quiet | 18 | 122.26 (119.18 to 125.34) |
| SBP | Previous hypertension diagnosis | Quiet | 21 | 121.42 (118.32 to 124.51) |
| SBP | Previous hypertension diagnosis | Quiet | 24 | 121.01 (117.90 to 124.12) |
| SBP | Previous hypertension diagnosis | Quiet | 27 | 122.00 (118.87 to 125.13) |
| SBP | Previous hypertension diagnosis | Quiet | 30 | 121.00 (117.85 to 124.15) |
| SBP | No previous hypertension diagnosis | Busy | 0 | 136.68 (133.61 to 139.75) |
| SBP | No previous hypertension diagnosis | Busy | 3 | 127.91 (124.84 to 130.98) |
| SBP | No previous hypertension diagnosis | Busy | 6 | 125.49 (122.41 to 128.56) |
| SBP | No previous hypertension diagnosis | Busy | 9 | 122.92 (119.84 to 126.00) |
| SBP | No previous hypertension diagnosis | Busy | 12 | 122.68 (119.59 to 125.77) |
| SBP | No previous hypertension diagnosis | Busy | 15 | 120.95 (117.85 to 124.05) |
| SBP | No previous hypertension diagnosis | Busy | 18 | 120.70 (117.59 to 123.81) |
| SBP | No previous hypertension diagnosis | Busy | 21 | 120.93 (117.80 to 124.05) |
| SBP | No previous hypertension diagnosis | Busy | 24 | 120.13 (116.99 to 123.27) |
| SBP | No previous hypertension diagnosis | Busy | 27 | 120.66 (117.50 to 123.82) |
| SBP | No previous hypertension diagnosis | Busy | 30 | 120.05 (116.87 to 123.23) |
| SBP | Previous hypertension diagnosis | Busy | 0 | 144.13 (141.09 to 147.17) |
| SBP | Previous hypertension diagnosis | Busy | 3 | 134.30 (131.26 to 137.34) |
| SBP | Previous hypertension diagnosis | Busy | 6 | 129.70 (126.65 to 132.74) |
| SBP | Previous hypertension diagnosis | Busy | 9 | 127.75 (124.70 to 130.80) |
| SBP | Previous hypertension diagnosis | Busy | 12 | 125.92 (122.86 to 128.98) |
| SBP | Previous hypertension diagnosis | Busy | 15 | 125.64 (122.58 to 128.71) |
| SBP | Previous hypertension diagnosis | Busy | 18 | 124.77 (121.69 to 127.85) |
| SBP | Previous hypertension diagnosis | Busy | 21 | 124.65 (121.56 to 127.75) |
| SBP | Previous hypertension diagnosis | Busy | 24 | 124.81 (121.70 to 127.92) |
| SBP | Previous hypertension diagnosis | Busy | 27 | 123.37 (120.24 to 126.50) |
| SBP | Previous hypertension diagnosis | Busy | 30 | 124.03 (120.87 to 127.18) |
| DBP | No previous hypertension diagnosis | Quiet | 0 | 83.20 (81.33 to 85.07) |
| DBP | No previous hypertension diagnosis | Quiet | 3 | 79.31 (77.44 to 81.18) |
| DBP | No previous hypertension diagnosis | Quiet | 6 | 77.51 (75.64 to 79.38) |
| DBP | No previous hypertension diagnosis | Quiet | 9 | 76.16 (74.28 to 78.03) |
| DBP | No previous hypertension diagnosis | Quiet | 12 | 75.61 (73.73 to 77.49) |
| DBP | No previous hypertension diagnosis | Quiet | 15 | 75.25 (73.36 to 77.14) |
| DBP | No previous hypertension diagnosis | Quiet | 18 | 75.33 (73.43 to 77.22) |
| DBP | No previous hypertension diagnosis | Quiet | 21 | 74.96 (73.06 to 76.87) |
| DBP | No previous hypertension diagnosis | Quiet | 24 | 74.30 (72.38 to 76.22) |
| DBP | No previous hypertension diagnosis | Quiet | 27 | 74.76 (72.83 to 76.69) |
| DBP | No previous hypertension diagnosis | Quiet | 30 | 74.58 (72.63 to 76.53) |
| DBP | Previous hypertension diagnosis | Quiet | 0 | 87.54 (85.69 to 89.39) |
| DBP | Previous hypertension diagnosis | Quiet | 3 | 84.10 (82.24 to 85.95) |
| DBP | Previous hypertension diagnosis | Quiet | 6 | 82.75 (80.90 to 84.60) |
| DBP | Previous hypertension diagnosis | Quiet | 9 | 81.94 (80.08 to 83.80) |
| DBP | Previous hypertension diagnosis | Quiet | 12 | 81.40 (79.53 to 83.26) |
| DBP | Previous hypertension diagnosis | Quiet | 15 | 80.69 (78.82 to 82.56) |
| DBP | Previous hypertension diagnosis | Quiet | 18 | 80.14 (78.26 to 82.02) |
| DBP | Previous hypertension diagnosis | Quiet | 21 | 80.29 (78.40 to 82.18) |
| DBP | Previous hypertension diagnosis | Quiet | 24 | 79.63 (77.73 to 81.53) |
| DBP | Previous hypertension diagnosis | Quiet | 27 | 80.33 (78.42 to 82.25) |
| DBP | Previous hypertension diagnosis | Quiet | 30 | 79.87 (77.94 to 81.80) |
| DBP | No previous hypertension diagnosis | Busy | 0 | 83.54 (81.67 to 85.41) |
| DBP | No previous hypertension diagnosis | Busy | 3 | 80.37 (78.50 to 82.24) |
| DBP | No previous hypertension diagnosis | Busy | 6 | 78.74 (76.87 to 80.61) |
| DBP | No previous hypertension diagnosis | Busy | 9 | 77.63 (75.76 to 79.51) |
| DBP | No previous hypertension diagnosis | Busy | 12 | 77.79 (75.91 to 79.67) |
| DBP | No previous hypertension diagnosis | Busy | 15 | 76.62 (74.73 to 78.51) |
| DBP | No previous hypertension diagnosis | Busy | 18 | 76.93 (75.04 to 78.83) |
| DBP | No previous hypertension diagnosis | Busy | 21 | 76.50 (74.59 to 78.40) |
| DBP | No previous hypertension diagnosis | Busy | 24 | 77.13 (75.21 to 79.05) |
| DBP | No previous hypertension diagnosis | Busy | 27 | 77.04 (75.11 to 78.97) |
| DBP | No previous hypertension diagnosis | Busy | 30 | 76.67 (74.73 to 78.62) |
| DBP | Previous hypertension diagnosis | Busy | 0 | 87.67 (85.82 to 89.52) |
| DBP | Previous hypertension diagnosis | Busy | 3 | 84.74 (82.89 to 86.59) |
| DBP | Previous hypertension diagnosis | Busy | 6 | 83.24 (81.38 to 85.09) |
| DBP | Previous hypertension diagnosis | Busy | 9 | 82.51 (80.65 to 84.36) |
| DBP | Previous hypertension diagnosis | Busy | 12 | 81.83 (79.96 to 83.69) |
| DBP | Previous hypertension diagnosis | Busy | 15 | 81.71 (79.84 to 83.58) |
| DBP | Previous hypertension diagnosis | Busy | 18 | 80.86 (78.98 to 82.74) |
| DBP | Previous hypertension diagnosis | Busy | 21 | 81.10 (79.21 to 82.99) |
| DBP | Previous hypertension diagnosis | Busy | 24 | 80.56 (78.66 to 82.46) |
| DBP | Previous hypertension diagnosis | Busy | 27 | 79.99 (78.07 to 81.90) |
| DBP | Previous hypertension diagnosis | Busy | 30 | 80.63 (78.70 to 82.56) |
| PR | No previous hypertension diagnosis | Quiet | 0 | 72.35 (70.06 to 74.64) |
| PR | No previous hypertension diagnosis | Quiet | 3 | 70.27 (67.98 to 72.56) |
| PR | No previous hypertension diagnosis | Quiet | 6 | 71.25 (68.96 to 73.54) |
| PR | No previous hypertension diagnosis | Quiet | 9 | 71.81 (69.52 to 74.10) |
| PR | No previous hypertension diagnosis | Quiet | 12 | 72.36 (70.06 to 74.65) |
| PR | No previous hypertension diagnosis | Quiet | 15 | 72.33 (70.03 to 74.63) |
| PR | No previous hypertension diagnosis | Quiet | 18 | 72.11 (69.81 to 74.42) |
| PR | No previous hypertension diagnosis | Quiet | 21 | 72.34 (70.03 to 74.64) |
| PR | No previous hypertension diagnosis | Quiet | 24 | 72.33 (70.02 to 74.64) |
| PR | No previous hypertension diagnosis | Quiet | 27 | 72.60 (70.28 to 74.92) |
| PR | No previous hypertension diagnosis | Quiet | 30 | 72.70 (70.37 to 75.02) |
| PR | Previous hypertension diagnosis | Quiet | 0 | 72.77 (70.50 to 75.03) |
| PR | Previous hypertension diagnosis | Quiet | 3 | 70.29 (68.02 to 72.55) |
| PR | Previous hypertension diagnosis | Quiet | 6 | 70.21 (67.94 to 72.48) |
| PR | Previous hypertension diagnosis | Quiet | 9 | 69.55 (67.28 to 71.82) |
| PR | Previous hypertension diagnosis | Quiet | 12 | 69.62 (67.35 to 71.90) |
| PR | Previous hypertension diagnosis | Quiet | 15 | 69.48 (67.21 to 71.76) |
| PR | Previous hypertension diagnosis | Quiet | 18 | 69.71 (67.43 to 71.99) |
| PR | Previous hypertension diagnosis | Quiet | 21 | 69.44 (67.16 to 71.73) |
| PR | Previous hypertension diagnosis | Quiet | 24 | 69.07 (66.77 to 71.36) |
| PR | Previous hypertension diagnosis | Quiet | 27 | 69.16 (66.86 to 71.45) |
| PR | Previous hypertension diagnosis | Quiet | 30 | 69.12 (66.82 to 71.43) |
| PR | No previous hypertension diagnosis | Busy | 0 | 75.13 (72.84 to 77.42) |
| PR | No previous hypertension diagnosis | Busy | 3 | 73.35 (71.06 to 75.64) |
| PR | No previous hypertension diagnosis | Busy | 6 | 74.15 (71.86 to 76.44) |
| PR | No previous hypertension diagnosis | Busy | 9 | 74.42 (72.13 to 76.72) |
| PR | No previous hypertension diagnosis | Busy | 12 | 74.53 (72.23 to 76.82) |
| PR | No previous hypertension diagnosis | Busy | 15 | 74.73 (72.43 to 77.03) |
| PR | No previous hypertension diagnosis | Busy | 18 | 74.58 (72.28 to 76.89) |
| PR | No previous hypertension diagnosis | Busy | 21 | 74.63 (72.33 to 76.94) |
| PR | No previous hypertension diagnosis | Busy | 24 | 74.58 (72.27 to 76.89) |
| PR | No previous hypertension diagnosis | Busy | 27 | 74.22 (71.91 to 76.54) |
| PR | No previous hypertension diagnosis | Busy | 30 | 74.57 (72.24 to 76.89) |
| PR | Previous hypertension diagnosis | Busy | 0 | 74.77 (72.51 to 77.04) |
| PR | Previous hypertension diagnosis | Busy | 3 | 71.89 (69.62 to 74.16) |
| PR | Previous hypertension diagnosis | Busy | 6 | 71.42 (69.15 to 73.69) |
| PR | Previous hypertension diagnosis | Busy | 9 | 71.40 (69.13 to 73.67) |
| PR | Previous hypertension diagnosis | Busy | 12 | 71.02 (68.74 to 73.29) |
| PR | Previous hypertension diagnosis | Busy | 15 | 70.82 (68.55 to 73.10) |
| PR | Previous hypertension diagnosis | Busy | 18 | 70.25 (67.97 to 72.53) |
| PR | Previous hypertension diagnosis | Busy | 21 | 70.50 (68.21 to 72.78) |
| PR | Previous hypertension diagnosis | Busy | 24 | 70.46 (68.17 to 72.75) |
| PR | Previous hypertension diagnosis | Busy | 27 | 70.25 (67.95 to 72.55) |
| PR | Previous hypertension diagnosis | Busy | 30 | 70.47 (68.17 to 72.78) |
| PP | No previous hypertension diagnosis | Quiet | 0 | 50.50 (48.30 to 52.70) |
| PP | No previous hypertension diagnosis | Quiet | 3 | 46.14 (43.94 to 48.34) |
| PP | No previous hypertension diagnosis | Quiet | 6 | 44.02 (41.81 to 46.22) |
| PP | No previous hypertension diagnosis | Quiet | 9 | 43.08 (40.88 to 45.29) |
| PP | No previous hypertension diagnosis | Quiet | 12 | 42.23 (40.02 to 44.44) |
| PP | No previous hypertension diagnosis | Quiet | 15 | 41.45 (39.24 to 43.67) |
| PP | No previous hypertension diagnosis | Quiet | 18 | 40.93 (38.71 to 43.15) |
| PP | No previous hypertension diagnosis | Quiet | 21 | 42.02 (39.79 to 44.26) |
| PP | No previous hypertension diagnosis | Quiet | 24 | 41.89 (39.65 to 44.13) |
| PP | No previous hypertension diagnosis | Quiet | 27 | 40.88 (38.63 to 43.13) |
| PP | No previous hypertension diagnosis | Quiet | 30 | 41.13 (38.87 to 43.40) |
| PP | Previous hypertension diagnosis | Quiet | 0 | 55.51 (53.33 to 57.69) |
| PP | Previous hypertension diagnosis | Quiet | 3 | 49.90 (47.72 to 52.08) |
| PP | Previous hypertension diagnosis | Quiet | 6 | 46.22 (44.04 to 48.40) |
| PP | Previous hypertension diagnosis | Quiet | 9 | 44.16 (41.97 to 46.35) |
| PP | Previous hypertension diagnosis | Quiet | 12 | 42.87 (40.68 to 45.06) |
| PP | Previous hypertension diagnosis | Quiet | 15 | 42.26 (40.07 to 44.46) |
| PP | Previous hypertension diagnosis | Quiet | 18 | 42.30 (40.10 to 44.51) |
| PP | Previous hypertension diagnosis | Quiet | 21 | 41.30 (39.09 to 43.51) |
| PP | Previous hypertension diagnosis | Quiet | 24 | 41.56 (39.34 to 43.78) |
| PP | Previous hypertension diagnosis | Quiet | 27 | 41.85 (39.62 to 44.08) |
| PP | Previous hypertension diagnosis | Quiet | 30 | 41.31 (39.07 to 43.56) |
| PP | No previous hypertension diagnosis | Busy | 0 | 52.95 (50.75 to 55.15) |
| PP | No previous hypertension diagnosis | Busy | 3 | 47.35 (45.15 to 49.55) |
| PP | No previous hypertension diagnosis | Busy | 6 | 46.56 (44.36 to 48.77) |
| PP | No previous hypertension diagnosis | Busy | 9 | 45.11 (42.90 to 47.31) |
| PP | No previous hypertension diagnosis | Busy | 12 | 44.70 (42.49 to 46.91) |
| PP | No previous hypertension diagnosis | Busy | 15 | 44.15 (41.93 to 46.37) |
| PP | No previous hypertension diagnosis | Busy | 18 | 43.58 (41.36 to 45.80) |
| PP | No previous hypertension diagnosis | Busy | 21 | 44.25 (42.02 to 46.48) |
| PP | No previous hypertension diagnosis | Busy | 24 | 42.82 (40.58 to 45.06) |
| PP | No previous hypertension diagnosis | Busy | 27 | 43.43 (41.18 to 45.68) |
| PP | No previous hypertension diagnosis | Busy | 30 | 43.19 (40.92 to 45.45) |
| PP | Previous hypertension diagnosis | Busy | 0 | 56.65 (54.47 to 58.83) |
| PP | Previous hypertension diagnosis | Busy | 3 | 49.74 (47.56 to 51.92) |
| PP | Previous hypertension diagnosis | Busy | 6 | 46.64 (44.46 to 48.83) |
| PP | Previous hypertension diagnosis | Busy | 9 | 45.43 (43.24 to 47.61) |
| PP | Previous hypertension diagnosis | Busy | 12 | 44.27 (42.08 to 46.47) |
| PP | Previous hypertension diagnosis | Busy | 15 | 44.12 (41.92 to 46.31) |
| PP | Previous hypertension diagnosis | Busy | 18 | 44.09 (41.89 to 46.29) |
| PP | Previous hypertension diagnosis | Busy | 21 | 43.73 (41.52 to 45.94) |
| PP | Previous hypertension diagnosis | Busy | 24 | 44.43 (42.21 to 46.65) |
| PP | Previous hypertension diagnosis | Busy | 27 | 43.56 (41.33 to 45.79) |
| PP | Previous hypertension diagnosis | Busy | 30 | 43.58 (41.33 to 45.82) |
| BP/pulse-pressure units: mmHg; pulse rate: beats/min. Common covariate averages and proportional weights for nuisance factors; pointwise 95% confidence intervals. These are model-based adjusted means, not the observed means in Figure 2. | | | | |

**Table S4. All environmental contrasts.**

| **Outcome** | **Group** | **Time (min)** | **Busy minus quiet (95% CI)** | **Nominal p** | **Holm p** |
| --- | --- | --- | --- | --- | --- |
| SBP | No previous hypertension diagnosis | 0 | 2.80 (1.43 to 4.16) | <0.001 | <0.001 |
| SBP | Previous hypertension diagnosis | 0 | 1.26 (-0.10 to 2.61) | 0.069 | 0.278 |
| SBP | No previous hypertension diagnosis | 3 | 2.27 (0.90 to 3.64) | 0.001 | 0.010 |
| SBP | Previous hypertension diagnosis | 3 | 0.48 (-0.88 to 1.85) | 0.484 | 0.484 |
| SBP | No previous hypertension diagnosis | 6 | 3.78 (2.40 to 5.17) | <0.001 | <0.001 |
| SBP | Previous hypertension diagnosis | 6 | 0.91 (-0.47 to 2.28) | 0.196 | 0.393 |
| SBP | No previous hypertension diagnosis | 9 | 3.50 (2.09 to 4.91) | <0.001 | <0.001 |
| SBP | Previous hypertension diagnosis | 9 | 1.83 (0.43 to 3.24) | 0.010 | 0.062 |
| SBP | No previous hypertension diagnosis | 12 | 4.66 (3.21 to 6.10) | <0.001 | <0.001 |
| SBP | Previous hypertension diagnosis | 12 | 1.83 (0.40 to 3.27) | 0.012 | 0.062 |
| SBP | No previous hypertension diagnosis | 15 | 4.07 (2.58 to 5.56) | <0.001 | <0.001 |
| SBP | Previous hypertension diagnosis | 15 | 2.87 (1.39 to 4.35) | <0.001 | 0.002 |
| SBP | No previous hypertension diagnosis | 18 | 4.26 (2.72 to 5.80) | <0.001 | <0.001 |
| SBP | Previous hypertension diagnosis | 18 | 2.51 (0.98 to 4.04) | 0.001 | 0.010 |
| SBP | No previous hypertension diagnosis | 21 | 3.76 (2.16 to 5.35) | <0.001 | <0.001 |
| SBP | Previous hypertension diagnosis | 21 | 3.24 (1.65 to 4.82) | <0.001 | <0.001 |
| SBP | No previous hypertension diagnosis | 24 | 3.75 (2.09 to 5.41) | <0.001 | <0.001 |
| SBP | Previous hypertension diagnosis | 24 | 3.80 (2.15 to 5.45) | <0.001 | <0.001 |
| SBP | No previous hypertension diagnosis | 27 | 4.84 (3.10 to 6.57) | <0.001 | <0.001 |
| SBP | Previous hypertension diagnosis | 27 | 1.37 (-0.35 to 3.09) | 0.119 | 0.356 |
| SBP | No previous hypertension diagnosis | 30 | 4.15 (2.34 to 5.96) | <0.001 | <0.001 |
| SBP | Previous hypertension diagnosis | 30 | 3.02 (1.23 to 4.82) | 0.001 | 0.009 |
| DBP | No previous hypertension diagnosis | 0 | 0.34 (-0.63 to 1.32) | 0.490 | 1.000 |
| DBP | Previous hypertension diagnosis | 0 | 0.12 (-0.84 to 1.09) | 0.803 | 1.000 |
| DBP | No previous hypertension diagnosis | 3 | 1.06 (0.08 to 2.04) | 0.033 | 0.433 |
| DBP | Previous hypertension diagnosis | 3 | 0.65 (-0.32 to 1.62) | 0.190 | 1.000 |
| DBP | No previous hypertension diagnosis | 6 | 1.23 (0.25 to 2.22) | 0.014 | 0.200 |
| DBP | Previous hypertension diagnosis | 6 | 0.49 (-0.49 to 1.46) | 0.330 | 1.000 |
| DBP | No previous hypertension diagnosis | 9 | 1.47 (0.47 to 2.47) | 0.004 | 0.068 |
| DBP | Previous hypertension diagnosis | 9 | 0.57 (-0.43 to 1.56) | 0.263 | 1.000 |
| DBP | No previous hypertension diagnosis | 12 | 2.18 (1.16 to 3.20) | <0.001 | <0.001 |
| DBP | Previous hypertension diagnosis | 12 | 0.43 (-0.58 to 1.45) | 0.403 | 1.000 |
| DBP | No previous hypertension diagnosis | 15 | 1.37 (0.32 to 2.42) | 0.011 | 0.158 |
| DBP | Previous hypertension diagnosis | 15 | 1.01 (-0.03 to 2.06) | 0.056 | 0.670 |
| DBP | No previous hypertension diagnosis | 18 | 1.61 (0.53 to 2.68) | 0.004 | 0.065 |
| DBP | Previous hypertension diagnosis | 18 | 0.72 (-0.35 to 1.79) | 0.188 | 1.000 |
| DBP | No previous hypertension diagnosis | 21 | 1.53 (0.42 to 2.65) | 0.007 | 0.114 |
| DBP | Previous hypertension diagnosis | 21 | 0.81 (-0.30 to 1.92) | 0.152 | 1.000 |
| DBP | No previous hypertension diagnosis | 24 | 2.83 (1.68 to 3.98) | <0.001 | <0.001 |
| DBP | Previous hypertension diagnosis | 24 | 0.93 (-0.22 to 2.07) | 0.113 | 1.000 |
| DBP | No previous hypertension diagnosis | 27 | 2.28 (1.09 to 3.48) | <0.001 | 0.004 |
| DBP | Previous hypertension diagnosis | 27 | -0.34 (-1.53 to 0.85) | 0.570 | 1.000 |
| DBP | No previous hypertension diagnosis | 30 | 2.09 (0.85 to 3.34) | 0.001 | 0.019 |
| DBP | Previous hypertension diagnosis | 30 | 0.76 (-0.48 to 1.99) | 0.228 | 1.000 |
| PR | No previous hypertension diagnosis | 0 | 2.79 (1.93 to 3.65) | <0.001 | <0.001 |
| PR | Previous hypertension diagnosis | 0 | 2.01 (1.15 to 2.86) | <0.001 | <0.001 |
| PR | No previous hypertension diagnosis | 3 | 3.08 (2.22 to 3.94) | <0.001 | <0.001 |
| PR | Previous hypertension diagnosis | 3 | 1.60 (0.75 to 2.46) | <0.001 | 0.003 |
| PR | No previous hypertension diagnosis | 6 | 2.90 (2.03 to 3.76) | <0.001 | <0.001 |
| PR | Previous hypertension diagnosis | 6 | 1.21 (0.35 to 2.07) | 0.006 | 0.030 |
| PR | No previous hypertension diagnosis | 9 | 2.61 (1.74 to 3.49) | <0.001 | <0.001 |
| PR | Previous hypertension diagnosis | 9 | 1.85 (0.98 to 2.72) | <0.001 | <0.001 |
| PR | No previous hypertension diagnosis | 12 | 2.17 (1.28 to 3.06) | <0.001 | <0.001 |
| PR | Previous hypertension diagnosis | 12 | 1.39 (0.51 to 2.28) | 0.002 | 0.017 |
| PR | No previous hypertension diagnosis | 15 | 2.40 (1.49 to 3.31) | <0.001 | <0.001 |
| PR | Previous hypertension diagnosis | 15 | 1.34 (0.44 to 2.24) | 0.004 | 0.026 |
| PR | No previous hypertension diagnosis | 18 | 2.47 (1.54 to 3.40) | <0.001 | <0.001 |
| PR | Previous hypertension diagnosis | 18 | 0.55 (-0.38 to 1.47) | 0.245 | 0.245 |
| PR | No previous hypertension diagnosis | 21 | 2.30 (1.34 to 3.25) | <0.001 | <0.001 |
| PR | Previous hypertension diagnosis | 21 | 1.05 (0.11 to 2.00) | 0.029 | 0.087 |
| PR | No previous hypertension diagnosis | 24 | 2.25 (1.27 to 3.23) | <0.001 | <0.001 |
| PR | Previous hypertension diagnosis | 24 | 1.39 (0.42 to 2.37) | 0.005 | 0.030 |
| PR | No previous hypertension diagnosis | 27 | 1.63 (0.62 to 2.63) | 0.002 | 0.014 |
| PR | Previous hypertension diagnosis | 27 | 1.09 (0.09 to 2.09) | 0.032 | 0.087 |
| PR | No previous hypertension diagnosis | 30 | 1.87 (0.83 to 2.91) | <0.001 | 0.004 |
| PR | Previous hypertension diagnosis | 30 | 1.35 (0.32 to 2.38) | 0.010 | 0.042 |
| PP | No previous hypertension diagnosis | 0 | 2.46 (1.12 to 3.79) | <0.001 | 0.006 |
| PP | Previous hypertension diagnosis | 0 | 1.13 (-0.20 to 2.46) | 0.094 | 0.391 |
| PP | No previous hypertension diagnosis | 3 | 1.21 (-0.13 to 2.55) | 0.078 | 0.391 |
| PP | Previous hypertension diagnosis | 3 | -0.16 (-1.49 to 1.17) | 0.811 | 1.000 |
| PP | No previous hypertension diagnosis | 6 | 2.55 (1.20 to 3.89) | <0.001 | 0.005 |
| PP | Previous hypertension diagnosis | 6 | 0.42 (-0.92 to 1.76) | 0.537 | 1.000 |
| PP | No previous hypertension diagnosis | 9 | 2.02 (0.67 to 3.38) | 0.004 | 0.046 |
| PP | Previous hypertension diagnosis | 9 | 1.27 (-0.08 to 2.62) | 0.065 | 0.391 |
| PP | No previous hypertension diagnosis | 12 | 2.47 (1.10 to 3.85) | <0.001 | 0.007 |
| PP | Previous hypertension diagnosis | 12 | 1.40 (0.04 to 2.77) | 0.044 | 0.306 |
| PP | No previous hypertension diagnosis | 15 | 2.70 (1.31 to 4.09) | <0.001 | 0.003 |
| PP | Previous hypertension diagnosis | 15 | 1.86 (0.47 to 3.24) | 0.009 | 0.095 |
| PP | No previous hypertension diagnosis | 18 | 2.65 (1.24 to 4.07) | <0.001 | 0.005 |
| PP | Previous hypertension diagnosis | 18 | 1.79 (0.38 to 3.19) | 0.013 | 0.114 |
| PP | No previous hypertension diagnosis | 21 | 2.22 (0.78 to 3.67) | 0.003 | 0.036 |
| PP | Previous hypertension diagnosis | 21 | 2.43 (1.00 to 3.86) | <0.001 | 0.015 |
| PP | No previous hypertension diagnosis | 24 | 0.92 (-0.55 to 2.40) | 0.217 | 0.652 |
| PP | Previous hypertension diagnosis | 24 | 2.87 (1.41 to 4.33) | <0.001 | 0.003 |
| PP | No previous hypertension diagnosis | 27 | 2.55 (1.05 to 4.06) | <0.001 | 0.015 |
| PP | Previous hypertension diagnosis | 27 | 1.71 (0.22 to 3.21) | 0.025 | 0.198 |
| PP | No previous hypertension diagnosis | 30 | 2.06 (0.52 to 3.60) | 0.009 | 0.095 |
| PP | Previous hypertension diagnosis | 30 | 2.26 (0.73 to 3.79) | 0.004 | 0.046 |
| Busy minus quiet; positive values indicate higher BP in the busy area. Units: SBP, DBP and pulse pressure, mmHg; pulse rate, beats/min. Reporting models: categorical time, environment, diagnostic group and all their interactions; age spline (3 df), sex, BMI, period and sequence; participant intercept, session intercept and slope with diagonal covariance, and within-session AR(1). Intervals are pointwise 95%, not multiplicity-adjusted. Complete time-specific nominal and Holm-adjusted p-values are supplied in Table S4. Selected times are a reporting subset, not a new prospective endpoint specification. Holm correction is within each model across 22 time-by-group contrasts, not across outcomes. | | | | | |

**Table S5. Adjusted comparisons between diagnostic groups.**

| **Outcome** | **Environment** | **Time (min)** | **Hypertension minus comparison (95% CI)** | **Nominal p** | **Holm p** |
| --- | --- | --- | --- | --- | --- |
| SBP | Quiet | 0 | 9.00 (3.91 to 14.09) | <0.001 | 0.012 |
| SBP | Busy | 0 | 7.45 (2.36 to 12.55) | 0.004 | 0.084 |
| SBP | Quiet | 3 | 8.18 (3.09 to 13.27) | 0.002 | 0.036 |
| SBP | Busy | 3 | 6.40 (1.31 to 11.49) | 0.014 | 0.237 |
| SBP | Quiet | 6 | 7.09 (1.99 to 12.18) | 0.007 | 0.124 |
| SBP | Busy | 6 | 4.21 (-0.89 to 9.31) | 0.105 | 0.736 |
| SBP | Quiet | 9 | 6.49 (1.39 to 11.60) | 0.013 | 0.230 |
| SBP | Busy | 9 | 4.83 (-0.28 to 9.93) | 0.064 | 0.700 |
| SBP | Quiet | 12 | 6.07 (0.95 to 11.18) | 0.020 | 0.319 |
| SBP | Busy | 12 | 3.24 (-1.87 to 8.36) | 0.213 | 0.736 |
| SBP | Quiet | 15 | 5.89 (0.76 to 11.01) | 0.024 | 0.342 |
| SBP | Busy | 15 | 4.69 (-0.43 to 9.82) | 0.073 | 0.726 |
| SBP | Quiet | 18 | 5.82 (0.68 to 10.96) | 0.026 | 0.344 |
| SBP | Busy | 18 | 4.07 (-1.07 to 9.21) | 0.120 | 0.736 |
| SBP | Quiet | 21 | 4.24 (-0.91 to 9.40) | 0.106 | 0.736 |
| SBP | Busy | 21 | 3.72 (-1.43 to 8.88) | 0.156 | 0.736 |
| SBP | Quiet | 24 | 4.64 (-0.54 to 9.81) | 0.079 | 0.726 |
| SBP | Busy | 24 | 4.68 (-0.50 to 9.86) | 0.076 | 0.726 |
| SBP | Quiet | 27 | 6.18 (0.98 to 11.38) | 0.020 | 0.319 |
| SBP | Busy | 27 | 2.72 (-2.49 to 7.92) | 0.305 | 0.736 |
| SBP | Quiet | 30 | 5.10 (-0.12 to 10.33) | 0.056 | 0.666 |
| SBP | Busy | 30 | 3.98 (-1.25 to 9.20) | 0.135 | 0.736 |
| DBP | Quiet | 0 | 4.34 (1.26 to 7.43) | 0.006 | 0.045 |
| DBP | Busy | 0 | 4.12 (1.04 to 7.21) | 0.009 | 0.054 |
| DBP | Quiet | 3 | 4.78 (1.70 to 7.87) | 0.002 | 0.030 |
| DBP | Busy | 3 | 4.37 (1.28 to 7.46) | 0.006 | 0.045 |
| DBP | Quiet | 6 | 5.24 (2.15 to 8.33) | <0.001 | 0.016 |
| DBP | Busy | 6 | 4.49 (1.40 to 7.58) | 0.004 | 0.041 |
| DBP | Quiet | 9 | 5.78 (2.68 to 8.87) | <0.001 | 0.006 |
| DBP | Busy | 9 | 4.87 (1.78 to 7.97) | 0.002 | 0.027 |
| DBP | Quiet | 12 | 5.79 (2.69 to 8.89) | <0.001 | 0.006 |
| DBP | Busy | 12 | 4.04 (0.93 to 7.14) | 0.011 | 0.054 |
| DBP | Quiet | 15 | 5.44 (2.33 to 8.55) | <0.001 | 0.012 |
| DBP | Busy | 15 | 5.09 (1.98 to 8.20) | 0.001 | 0.020 |
| DBP | Quiet | 18 | 4.81 (1.69 to 7.93) | 0.003 | 0.030 |
| DBP | Busy | 18 | 3.93 (0.81 to 7.05) | 0.014 | 0.055 |
| DBP | Quiet | 21 | 5.33 (2.20 to 8.46) | <0.001 | 0.016 |
| DBP | Busy | 21 | 4.61 (1.47 to 7.74) | 0.004 | 0.041 |
| DBP | Quiet | 24 | 5.33 (2.19 to 8.48) | <0.001 | 0.016 |
| DBP | Busy | 24 | 3.43 (0.28 to 6.58) | 0.033 | 0.065 |
| DBP | Quiet | 27 | 5.57 (2.41 to 8.74) | <0.001 | 0.012 |
| DBP | Busy | 27 | 2.95 (-0.22 to 6.11) | 0.068 | 0.068 |
| DBP | Quiet | 30 | 5.29 (2.11 to 8.47) | 0.001 | 0.018 |
| DBP | Busy | 30 | 3.96 (0.78 to 7.14) | 0.015 | 0.055 |
| PR | Quiet | 0 | 0.42 (-3.39 to 4.23) | 0.829 | 1.000 |
| PR | Busy | 0 | -0.36 (-4.17 to 3.45) | 0.853 | 1.000 |
| PR | Quiet | 3 | 0.01 (-3.80 to 3.82) | 0.995 | 1.000 |
| PR | Busy | 3 | -1.46 (-5.27 to 2.35) | 0.451 | 1.000 |
| PR | Quiet | 6 | -1.04 (-4.85 to 2.77) | 0.591 | 1.000 |
| PR | Busy | 6 | -2.73 (-6.54 to 1.08) | 0.160 | 1.000 |
| PR | Quiet | 9 | -2.26 (-6.07 to 1.55) | 0.245 | 1.000 |
| PR | Busy | 9 | -3.03 (-6.84 to 0.79) | 0.119 | 1.000 |
| PR | Quiet | 12 | -2.73 (-6.55 to 1.08) | 0.160 | 1.000 |
| PR | Busy | 12 | -3.51 (-7.33 to 0.30) | 0.071 | 1.000 |
| PR | Quiet | 15 | -2.85 (-6.66 to 0.97) | 0.144 | 1.000 |
| PR | Busy | 15 | -3.91 (-7.73 to -0.09) | 0.045 | 0.769 |
| PR | Quiet | 18 | -2.41 (-6.23 to 1.42) | 0.217 | 1.000 |
| PR | Busy | 18 | -4.33 (-8.15 to -0.51) | 0.027 | 0.585 |
| PR | Quiet | 21 | -2.89 (-6.72 to 0.94) | 0.138 | 1.000 |
| PR | Busy | 21 | -4.14 (-7.97 to -0.31) | 0.034 | 0.720 |
| PR | Quiet | 24 | -3.26 (-7.10 to 0.57) | 0.095 | 1.000 |
| PR | Busy | 24 | -4.12 (-7.96 to -0.28) | 0.035 | 0.720 |
| PR | Quiet | 27 | -3.44 (-7.28 to 0.40) | 0.079 | 1.000 |
| PR | Busy | 27 | -3.98 (-7.82 to -0.13) | 0.043 | 0.769 |
| PR | Quiet | 30 | -3.57 (-7.42 to 0.28) | 0.069 | 1.000 |
| PR | Busy | 30 | -4.09 (-7.94 to -0.24) | 0.037 | 0.720 |
| PP | Quiet | 0 | 5.02 (1.41 to 8.62) | 0.007 | 0.144 |
| PP | Busy | 0 | 3.69 (0.09 to 7.30) | 0.045 | 0.896 |
| PP | Quiet | 3 | 3.76 (0.15 to 7.37) | 0.041 | 0.864 |
| PP | Busy | 3 | 2.39 (-1.22 to 6.00) | 0.193 | 1.000 |
| PP | Quiet | 6 | 2.21 (-1.40 to 5.82) | 0.230 | 1.000 |
| PP | Busy | 6 | 0.08 (-3.53 to 3.69) | 0.965 | 1.000 |
| PP | Quiet | 9 | 1.08 (-2.54 to 4.69) | 0.559 | 1.000 |
| PP | Busy | 9 | 0.32 (-3.30 to 3.93) | 0.862 | 1.000 |
| PP | Quiet | 12 | 0.64 (-2.98 to 4.26) | 0.727 | 1.000 |
| PP | Busy | 12 | -0.43 (-4.05 to 3.19) | 0.816 | 1.000 |
| PP | Quiet | 15 | 0.81 (-2.82 to 4.44) | 0.661 | 1.000 |
| PP | Busy | 15 | -0.03 (-3.66 to 3.60) | 0.987 | 1.000 |
| PP | Quiet | 18 | 1.37 (-2.26 to 5.01) | 0.458 | 1.000 |
| PP | Busy | 18 | 0.51 (-3.13 to 4.15) | 0.783 | 1.000 |
| PP | Quiet | 21 | -0.72 (-4.37 to 2.92) | 0.697 | 1.000 |
| PP | Busy | 21 | -0.52 (-4.16 to 3.13) | 0.781 | 1.000 |
| PP | Quiet | 24 | -0.33 (-3.99 to 3.32) | 0.857 | 1.000 |
| PP | Busy | 24 | 1.61 (-2.04 to 5.27) | 0.386 | 1.000 |
| PP | Quiet | 27 | 0.97 (-2.70 to 4.64) | 0.604 | 1.000 |
| PP | Busy | 27 | 0.13 (-3.54 to 3.80) | 0.944 | 1.000 |
| PP | Quiet | 30 | 0.18 (-3.51 to 3.86) | 0.924 | 1.000 |
| PP | Busy | 30 | 0.39 (-3.30 to 4.07) | 0.837 | 1.000 |
| Hypertension minus participants without a previous diagnosis; these observational group comparisons are not causal effects. BP/PP in mmHg, pulse rate in beats/min. Pointwise 95% intervals. Holm p-values account for 22 time-by-environment contrasts within each model. | | | | | |

**Table S6. Period, sequence and time-by-period tests.**

| **Model** | **Test** | **Numerator df** | **Denominator df** | **Statistic** | **Nominal p** |
| --- | --- | --- | --- | --- | --- |
| SBP | period | 1 | 440 | 0.094 | 0.760 |
| SBP | sequence | 1 | 435 | 3.411 | 0.065 |
| DBP | period | 1 | 440 | 1.955 | 0.163 |
| DBP | sequence | 1 | 435 | 5.234 | 0.023 |
| PR | period | 1 | 440 | 0.235 | 0.628 |
| PR | sequence | 1 | 435 | 0.255 | 0.614 |
| PP | period | 1 | 440 | 0.350 | 0.554 |
| PP | sequence | 1 | 435 | 0.564 | 0.453 |
| SBP time-by-period sensitivity | period_main_term | 1 | 440 | 0.103 | 0.748 |
| SBP time-by-period sensitivity | period_by_time | 10 | 8810 | 0.571 | 0.839 |
| Entries are F-tests with their original denominator degrees of freedom. All p-values are nominal, exploratory tests. The period main term in the interaction model depends on the fitted contrast coding. The supplementary joint Wald chi-square is 5.800 with 11 df, p = 0.886 ; it is asymptotic and not the failed combined F-test. None of these tests certifies absence of carryover. A DBP sequence association must not be reported as proof of carryover. | | | | | |

**Table S7. Selected SBP sensitivity contrasts.**

| **Model** | **Group** | **Time (min)** | **Busy minus quiet SBP (95% CI)** | **Nominal p** |
| --- | --- | --- | --- | --- |
| Participant intercept | No previous hypertension diagnosis | 0 | 2.80 (1.48 to 4.12) | <0.001 |
| Participant intercept | Previous hypertension diagnosis | 0 | 1.26 (-0.06 to 2.57) | 0.061 |
| Participant intercept | No previous hypertension diagnosis | 6 | 3.78 (2.46 to 5.10) | <0.001 |
| Participant intercept | Previous hypertension diagnosis | 6 | 0.91 (-0.41 to 2.22) | 0.176 |
| Participant intercept | No previous hypertension diagnosis | 12 | 4.66 (3.34 to 5.98) | <0.001 |
| Participant intercept | Previous hypertension diagnosis | 12 | 1.83 (0.52 to 3.15) | 0.006 |
| Participant intercept | No previous hypertension diagnosis | 15 | 4.07 (2.75 to 5.39) | <0.001 |
| Participant intercept | Previous hypertension diagnosis | 15 | 2.87 (1.56 to 4.18) | <0.001 |
| Participant intercept | No previous hypertension diagnosis | 30 | 4.15 (2.83 to 5.47) | <0.001 |
| Participant intercept | Previous hypertension diagnosis | 30 | 3.02 (1.71 to 4.33) | <0.001 |
| Session intercept | No previous hypertension diagnosis | 0 | 2.80 (1.37 to 4.23) | <0.001 |
| Session intercept | Previous hypertension diagnosis | 0 | 1.26 (-0.17 to 2.68) | 0.083 |
| Session intercept | No previous hypertension diagnosis | 6 | 3.78 (2.35 to 5.21) | <0.001 |
| Session intercept | Previous hypertension diagnosis | 6 | 0.91 (-0.52 to 2.33) | 0.211 |
| Session intercept | No previous hypertension diagnosis | 12 | 4.66 (3.23 to 6.09) | <0.001 |
| Session intercept | Previous hypertension diagnosis | 12 | 1.83 (0.41 to 3.26) | 0.012 |
| Session intercept | No previous hypertension diagnosis | 15 | 4.07 (2.63 to 5.50) | <0.001 |
| Session intercept | Previous hypertension diagnosis | 15 | 2.87 (1.45 to 4.29) | <0.001 |
| Session intercept | No previous hypertension diagnosis | 30 | 4.15 (2.72 to 5.58) | <0.001 |
| Session intercept | Previous hypertension diagnosis | 30 | 3.02 (1.60 to 4.45) | <0.001 |
| Intercepts + AR(1) | No previous hypertension diagnosis | 0 | 2.80 (1.34 to 4.26) | <0.001 |
| Intercepts + AR(1) | Previous hypertension diagnosis | 0 | 1.26 (-0.19 to 2.70) | 0.089 |
| Intercepts + AR(1) | No previous hypertension diagnosis | 6 | 3.78 (2.32 to 5.24) | <0.001 |
| Intercepts + AR(1) | Previous hypertension diagnosis | 6 | 0.91 (-0.54 to 2.35) | 0.219 |
| Intercepts + AR(1) | No previous hypertension diagnosis | 12 | 4.66 (3.20 to 6.12) | <0.001 |
| Intercepts + AR(1) | Previous hypertension diagnosis | 12 | 1.83 (0.39 to 3.28) | 0.013 |
| Intercepts + AR(1) | No previous hypertension diagnosis | 15 | 4.07 (2.61 to 5.52) | <0.001 |
| Intercepts + AR(1) | Previous hypertension diagnosis | 15 | 2.87 (1.42 to 4.32) | <0.001 |
| Intercepts + AR(1) | No previous hypertension diagnosis | 30 | 4.15 (2.69 to 5.61) | <0.001 |
| Intercepts + AR(1) | Previous hypertension diagnosis | 30 | 3.02 (1.58 to 4.47) | <0.001 |
| Reporting model | No previous hypertension diagnosis | 0 | 2.80 (1.43 to 4.16) | <0.001 |
| Reporting model | Previous hypertension diagnosis | 0 | 1.26 (-0.10 to 2.61) | 0.069 |
| Reporting model | No previous hypertension diagnosis | 6 | 3.78 (2.40 to 5.17) | <0.001 |
| Reporting model | Previous hypertension diagnosis | 6 | 0.91 (-0.47 to 2.28) | 0.196 |
| Reporting model | No previous hypertension diagnosis | 12 | 4.66 (3.21 to 6.10) | <0.001 |
| Reporting model | Previous hypertension diagnosis | 12 | 1.83 (0.40 to 3.27) | 0.012 |
| Reporting model | No previous hypertension diagnosis | 15 | 4.07 (2.58 to 5.56) | <0.001 |
| Reporting model | Previous hypertension diagnosis | 15 | 2.87 (1.39 to 4.35) | <0.001 |
| Reporting model | No previous hypertension diagnosis | 30 | 4.15 (2.34 to 5.96) | <0.001 |
| Reporting model | Previous hypertension diagnosis | 30 | 3.02 (1.23 to 4.82) | 0.001 |
| Time-varying covariates | No previous hypertension diagnosis | 0 | 2.80 (1.44 to 4.16) | <0.001 |
| Time-varying covariates | Previous hypertension diagnosis | 0 | 1.26 (-0.09 to 2.61) | 0.068 |
| Time-varying covariates | No previous hypertension diagnosis | 6 | 3.78 (2.40 to 5.16) | <0.001 |
| Time-varying covariates | Previous hypertension diagnosis | 6 | 0.91 (-0.46 to 2.27) | 0.193 |
| Time-varying covariates | No previous hypertension diagnosis | 12 | 4.66 (3.23 to 6.09) | <0.001 |
| Time-varying covariates | Previous hypertension diagnosis | 12 | 1.83 (0.41 to 3.25) | 0.011 |
| Time-varying covariates | No previous hypertension diagnosis | 15 | 4.07 (2.60 to 5.53) | <0.001 |
| Time-varying covariates | Previous hypertension diagnosis | 15 | 2.87 (1.41 to 4.33) | <0.001 |
| Time-varying covariates | No previous hypertension diagnosis | 30 | 4.15 (2.40 to 5.90) | <0.001 |
| Time-varying covariates | Previous hypertension diagnosis | 30 | 3.02 (1.28 to 4.76) | <0.001 |
| Age overlap (25-79 y) | No previous hypertension diagnosis | 0 | 3.15 (1.65 to 4.64) | <0.001 |
| Age overlap (25-79 y) | Previous hypertension diagnosis | 0 | 0.97 (-0.40 to 2.34) | 0.166 |
| Age overlap (25-79 y) | No previous hypertension diagnosis | 6 | 3.90 (2.38 to 5.41) | <0.001 |
| Age overlap (25-79 y) | Previous hypertension diagnosis | 6 | 0.94 (-0.45 to 2.33) | 0.184 |
| Age overlap (25-79 y) | No previous hypertension diagnosis | 12 | 4.80 (3.23 to 6.37) | <0.001 |
| Age overlap (25-79 y) | Previous hypertension diagnosis | 12 | 1.84 (0.40 to 3.29) | 0.012 |
| Age overlap (25-79 y) | No previous hypertension diagnosis | 15 | 4.30 (2.68 to 5.91) | <0.001 |
| Age overlap (25-79 y) | Previous hypertension diagnosis | 15 | 2.88 (1.39 to 4.36) | <0.001 |
| Age overlap (25-79 y) | No previous hypertension diagnosis | 30 | 4.63 (2.69 to 6.56) | <0.001 |
| Age overlap (25-79 y) | Previous hypertension diagnosis | 30 | 3.18 (1.40 to 4.96) | <0.001 |
| Time-by-period model | No previous hypertension diagnosis | 0 | 2.80 (1.43 to 4.17) | <0.001 |
| Time-by-period model | Previous hypertension diagnosis | 0 | 1.26 (-0.10 to 2.61) | 0.070 |
| Time-by-period model | No previous hypertension diagnosis | 6 | 3.78 (2.40 to 5.17) | <0.001 |
| Time-by-period model | Previous hypertension diagnosis | 6 | 0.91 (-0.47 to 2.28) | 0.197 |
| Time-by-period model | No previous hypertension diagnosis | 12 | 4.66 (3.21 to 6.10) | <0.001 |
| Time-by-period model | Previous hypertension diagnosis | 12 | 1.84 (0.40 to 3.27) | 0.012 |
| Time-by-period model | No previous hypertension diagnosis | 15 | 4.06 (2.57 to 5.55) | <0.001 |
| Time-by-period model | Previous hypertension diagnosis | 15 | 2.87 (1.39 to 4.35) | <0.001 |
| Time-by-period model | No previous hypertension diagnosis | 30 | 4.15 (2.35 to 5.96) | <0.001 |
| Time-by-period model | Previous hypertension diagnosis | 30 | 3.02 (1.23 to 4.82) | 0.001 |
| First period only | No previous hypertension diagnosis | 0 | 3.62 (-1.15 to 8.40) | 0.137 |
| First period only | Previous hypertension diagnosis | 0 | 6.36 (1.61 to 11.12) | 0.009 |
| First period only | No previous hypertension diagnosis | 6 | 5.73 (0.96 to 10.51) | 0.019 |
| First period only | Previous hypertension diagnosis | 6 | 5.04 (0.28 to 9.80) | 0.038 |
| First period only | No previous hypertension diagnosis | 12 | 5.90 (1.12 to 10.68) | 0.016 |
| First period only | Previous hypertension diagnosis | 12 | 6.11 (1.35 to 10.87) | 0.012 |
| First period only | No previous hypertension diagnosis | 15 | 5.83 (1.05 to 10.60) | 0.017 |
| First period only | Previous hypertension diagnosis | 15 | 7.22 (2.46 to 11.98) | 0.003 |
| First period only | No previous hypertension diagnosis | 30 | 5.67 (0.89 to 10.44) | 0.020 |
| First period only | Previous hypertension diagnosis | 30 | 7.13 (2.37 to 11.89) | 0.003 |
| Cluster-robust OLS | No previous hypertension diagnosis | 0 | 2.80 (1.14 to 4.46) | 0.001 |
| Cluster-robust OLS | Previous hypertension diagnosis | 0 | 1.26 (-0.45 to 2.96) | 0.149 |
| Cluster-robust OLS | No previous hypertension diagnosis | 6 | 3.78 (2.57 to 4.99) | <0.001 |
| Cluster-robust OLS | Previous hypertension diagnosis | 6 | 0.91 (-0.57 to 2.38) | 0.228 |
| Cluster-robust OLS | No previous hypertension diagnosis | 12 | 4.66 (3.45 to 5.86) | <0.001 |
| Cluster-robust OLS | Previous hypertension diagnosis | 12 | 1.83 (0.53 to 3.14) | 0.006 |
| Cluster-robust OLS | No previous hypertension diagnosis | 15 | 4.07 (2.92 to 5.22) | <0.001 |
| Cluster-robust OLS | Previous hypertension diagnosis | 15 | 2.87 (1.67 to 4.07) | <0.001 |
| Cluster-robust OLS | No previous hypertension diagnosis | 30 | 4.15 (2.84 to 5.46) | <0.001 |
| Cluster-robust OLS | Previous hypertension diagnosis | 30 | 3.02 (1.69 to 4.36) | <0.001 |
| All periods: n=443; age-overlap sensitivity: n=400; first period only: n=443 with one environment per participant. Time-varying-covariate models interact age-spline terms, sex and BMI with categorical time. First-period analyses omit sequence and period because they are not separately identifiable within period 1. Intervals are pointwise; different point estimates should be reported, not described as identical. | | | | |

**Table S8. Joint trajectory tests.**

| **Model** | **Test** | **Numerator df** | **Denominator df** | **F** | **Nominal p** |
| --- | --- | --- | --- | --- | --- |
| Participant intercept | group_trajectory | 20 | 9260 | 4.291 | <0.001 |
| Participant intercept | environmental_trajectory | 20 | 9260 | 1.875 | 0.010 |
| Participant intercept | period_terms | 1 | 9260 | 0.683 | 0.409 |
| Session intercept | group_trajectory | 20 | 8820 | 5.274 | <0.001 |
| Session intercept | environmental_trajectory | 20 | 8820 | 2.304 | <0.001 |
| Session intercept | period_terms | 1 | 440 | 0.144 | 0.704 |
| Intercepts + AR(1) | group_trajectory | 20 | 8820 | 3.945 | <0.001 |
| Intercepts + AR(1) | environmental_trajectory | 20 | 8820 | 2.059 | 0.004 |
| Intercepts + AR(1) | period_terms | 1 | 440 | 0.056 | 0.813 |
| SBP reporting | group_trajectory | 20 | 8820 | 3.779 | <0.001 |
| SBP reporting | environmental_trajectory | 20 | 8820 | 2.021 | 0.004 |
| SBP reporting | period_terms | 1 | 440 | 0.094 | 0.760 |
| Time-varying covariates | group_trajectory | 20 | 8770 | 1.482 | 0.076 |
| Time-varying covariates | environmental_trajectory | 20 | 8770 | 2.057 | 0.004 |
| Time-varying covariates | period_terms | 1 | 440 | 0.095 | 0.758 |
| Age overlap (25-79 y) | group_trajectory | 20 | 7910 | 1.380 | 0.120 |
| Age overlap (25-79 y) | environmental_trajectory | 20 | 7910 | 1.919 | 0.008 |
| Age overlap (25-79 y) | period_terms | 1 | 397 | 0.745 | 0.389 |
| First period only | group_trajectory | 20 | 4390 | 1.739 | 0.022 |
| First period only | environmental_trajectory | 20 | 4390 | 1.191 | 0.251 |
| DBP reporting | group_trajectory | 20 | 8820 | 1.588 | 0.046 |
| DBP reporting | environmental_trajectory | 20 | 8820 | 1.506 | 0.068 |
| DBP reporting | period_terms | 1 | 440 | 1.955 | 0.163 |
| PR reporting | group_trajectory | 20 | 8820 | 6.625 | <0.001 |
| PR reporting | environmental_trajectory | 20 | 8820 | 0.893 | 0.596 |
| PR reporting | period_terms | 1 | 440 | 0.235 | 0.628 |
| PP reporting | group_trajectory | 20 | 8820 | 4.731 | <0.001 |
| PP reporting | environmental_trajectory | 20 | 8820 | 1.331 | 0.146 |
| PP reporting | period_terms | 1 | 440 | 0.350 | 0.554 |
| Time-by-period model | group_trajectory | 20 | 8810 | 3.776 | <0.001 |
| Time-by-period model | environmental_trajectory | 20 | 8810 | 2.020 | 0.005 |
| Time-by-period model | period_main_term | 1 | 440 | 0.103 | 0.748 |
| Time-by-period model | period_by_time | 10 | 8810 | 0.571 | 0.839 |
| Group trajectory jointly tests time-by-group plus time-by-environment-by-group terms. Environmental trajectory jointly tests time-by-environment plus time-by-environment-by-group terms. Repaired period main and period-by-time terms are listed separately. Nominal p-values; lower-order interaction tests alone do not summarize the three-way interaction model. | | | | | |

**Table S9a. Full disagreement trajectory.**

| **Group** | **Environment** | **Time (min)** | **Disagreement n/N; % (95% CI)** |
| --- | --- | --- | --- |
| Previous hypertension diagnosis | Busy | 0 | 84/223; 37.7% (31.6 to 44.2) |
| Previous hypertension diagnosis | Busy | 3 | 58/223; 26.0% (20.7 to 32.1) |
| Previous hypertension diagnosis | Busy | 6 | 40/223; 17.9% (13.5 to 23.5) |
| Previous hypertension diagnosis | Busy | 9 | 30/223; 13.5% (9.6 to 18.6) |
| Previous hypertension diagnosis | Busy | 12 | 24/223; 10.8% (7.3 to 15.5) |
| Previous hypertension diagnosis | Busy | 15 | 17/223; 7.6% (4.8 to 11.9) |
| Previous hypertension diagnosis | Busy | 18 | 28/223; 12.6% (8.8 to 17.5) |
| Previous hypertension diagnosis | Busy | 21 | 26/223; 11.7% (8.1 to 16.5) |
| Previous hypertension diagnosis | Busy | 24 | 20/223; 9.0% (5.9 to 13.4) |
| Previous hypertension diagnosis | Busy | 27 | 18/223; 8.1% (5.2 to 12.4) |
| Previous hypertension diagnosis | Busy | 30 | 18/223; 8.1% (5.2 to 12.4) |
| Previous hypertension diagnosis | Quiet | 0 | 90/223; 40.4% (34.1 to 46.9) |
| Previous hypertension diagnosis | Quiet | 3 | 65/223; 29.1% (23.6 to 35.4) |
| Previous hypertension diagnosis | Quiet | 6 | 44/223; 19.7% (15.0 to 25.4) |
| Previous hypertension diagnosis | Quiet | 9 | 33/223; 14.8% (10.7 to 20.1) |
| Previous hypertension diagnosis | Quiet | 12 | 28/223; 12.6% (8.8 to 17.5) |
| Previous hypertension diagnosis | Quiet | 15 | 24/223; 10.8% (7.3 to 15.5) |
| Previous hypertension diagnosis | Quiet | 18 | 23/223; 10.3% (7.0 to 15.0) |
| Previous hypertension diagnosis | Quiet | 21 | 19/223; 8.5% (5.5 to 12.9) |
| Previous hypertension diagnosis | Quiet | 24 | 13/223; 5.8% (3.4 to 9.7) |
| Previous hypertension diagnosis | Quiet | 27 | 9/223; 4.0% (2.1 to 7.5) |
| Previous hypertension diagnosis | Quiet | 30 | 18/223; 8.1% (5.2 to 12.4) |
| No previous hypertension diagnosis | Busy | 0 | 57/220; 25.9% (20.6 to 32.1) |
| No previous hypertension diagnosis | Busy | 3 | 26/220; 11.8% (8.2 to 16.8) |
| No previous hypertension diagnosis | Busy | 6 | 25/220; 11.4% (7.8 to 16.2) |
| No previous hypertension diagnosis | Busy | 9 | 19/220; 8.6% (5.6 to 13.1) |
| No previous hypertension diagnosis | Busy | 12 | 15/220; 6.8% (4.2 to 10.9) |
| No previous hypertension diagnosis | Busy | 15 | 13/220; 5.9% (3.5 to 9.8) |
| No previous hypertension diagnosis | Busy | 18 | 15/220; 6.8% (4.2 to 10.9) |
| No previous hypertension diagnosis | Busy | 21 | 18/220; 8.2% (5.2 to 12.6) |
| No previous hypertension diagnosis | Busy | 24 | 10/220; 4.5% (2.5 to 8.2) |
| No previous hypertension diagnosis | Busy | 27 | 11/220; 5.0% (2.8 to 8.7) |
| No previous hypertension diagnosis | Busy | 30 | 11/220; 5.0% (2.8 to 8.7) |
| No previous hypertension diagnosis | Quiet | 0 | 53/220; 24.1% (18.9 to 30.2) |
| No previous hypertension diagnosis | Quiet | 3 | 25/220; 11.4% (7.8 to 16.2) |
| No previous hypertension diagnosis | Quiet | 6 | 17/220; 7.7% (4.9 to 12.0) |
| No previous hypertension diagnosis | Quiet | 9 | 6/220; 2.7% (1.3 to 5.8) |
| No previous hypertension diagnosis | Quiet | 12 | 14/220; 6.4% (3.8 to 10.4) |
| No previous hypertension diagnosis | Quiet | 15 | 5/220; 2.3% (1.0 to 5.2) |
| No previous hypertension diagnosis | Quiet | 18 | 11/220; 5.0% (2.8 to 8.7) |
| No previous hypertension diagnosis | Quiet | 21 | 9/220; 4.1% (2.2 to 7.6) |
| No previous hypertension diagnosis | Quiet | 24 | 9/220; 4.1% (2.2 to 7.6) |
| No previous hypertension diagnosis | Quiet | 27 | 8/220; 3.6% (1.9 to 7.0) |
| No previous hypertension diagnosis | Quiet | 30 | 7/220; 3.2% (1.5 to 6.4) |
| Pointwise Wilson intervals, as defined in Figure 3. Both directions of threshold disagreement are included. | | | |

**Table S9b. Paired environmental comparisons of disagreement.**

| **Group** | **Time (min)** | **Pairs** | **Busy-only / quiet-only discordance** | **Difference, pp (95% CI)** | **Exact McNemar p** | **Holm p** |
| --- | --- | --- | --- | --- | --- | --- |
| No previous hypertension diagnosis | 0 | 220 | 26 / 22 | 1.82 (-4.55 to 8.18) | 0.665 | 1.000 |
| No previous hypertension diagnosis | 3 | 220 | 18 / 17 | 0.45 (-5.00 to 5.91) | 1.000 | 1.000 |
| No previous hypertension diagnosis | 6 | 220 | 18 / 10 | 3.64 (-0.91 to 8.18) | 0.185 | 1.000 |
| No previous hypertension diagnosis | 9 | 220 | 16 / 3 | 5.91 (2.27 to 10.00) | 0.004 | 0.097 |
| No previous hypertension diagnosis | 12 | 220 | 13 / 12 | 0.45 (-4.09 to 5.00) | 1.000 | 1.000 |
| No previous hypertension diagnosis | 15 | 220 | 12 / 4 | 3.64 (0.45 to 7.27) | 0.077 | 1.000 |
| No previous hypertension diagnosis | 18 | 220 | 11 / 7 | 1.82 (-1.82 to 5.45) | 0.481 | 1.000 |
| No previous hypertension diagnosis | 21 | 220 | 13 / 4 | 4.09 (0.45 to 7.73) | 0.049 | 1.000 |
| No previous hypertension diagnosis | 24 | 220 | 7 / 6 | 0.45 (-2.73 to 3.64) | 1.000 | 1.000 |
| No previous hypertension diagnosis | 27 | 220 | 11 / 8 | 1.36 (-2.73 to 5.45) | 0.648 | 1.000 |
| No previous hypertension diagnosis | 30 | 220 | 10 / 6 | 1.82 (-1.82 to 5.45) | 0.454 | 1.000 |
| Previous hypertension diagnosis | 0 | 223 | 26 / 32 | -2.69 (-9.42 to 4.04) | 0.512 | 1.000 |
| Previous hypertension diagnosis | 3 | 223 | 28 / 35 | -3.14 (-10.31 to 4.04) | 0.450 | 1.000 |
| Previous hypertension diagnosis | 6 | 223 | 25 / 29 | -1.79 (-8.07 to 4.48) | 0.683 | 1.000 |
| Previous hypertension diagnosis | 9 | 223 | 24 / 27 | -1.35 (-7.62 to 4.93) | 0.780 | 1.000 |
| Previous hypertension diagnosis | 12 | 223 | 16 / 20 | -1.79 (-6.73 to 3.14) | 0.618 | 1.000 |
| Previous hypertension diagnosis | 15 | 223 | 10 / 17 | -3.14 (-7.62 to 1.35) | 0.248 | 1.000 |
| Previous hypertension diagnosis | 18 | 223 | 20 / 15 | 2.24 (-2.69 to 7.62) | 0.500 | 1.000 |
| Previous hypertension diagnosis | 21 | 223 | 24 / 17 | 3.14 (-2.24 to 8.97) | 0.349 | 1.000 |
| Previous hypertension diagnosis | 24 | 223 | 18 / 11 | 3.14 (-1.79 to 8.07) | 0.265 | 1.000 |
| Previous hypertension diagnosis | 27 | 223 | 17 / 8 | 4.04 (-0.45 to 8.52) | 0.108 | 1.000 |
| Previous hypertension diagnosis | 30 | 223 | 13 / 13 | 0.00 (-4.48 to 4.48) | 1.000 | 1.000 |
| pp denotes percentage points; difference is busy minus quiet. Confidence intervals are pointwise participant-pair percentile-bootstrap intervals; p-values use the separate exact McNemar test. Their significance boundaries need not coincide, particularly with few discordant pairs. Do not use a bootstrap interval to relabel the exact p-value. Holm adjustment is across all 22 group-by-time paired tests. These descriptive paired tests do not separately adjust for period/sequence. | | | | | | |

**Table S10a. Late-session stability and elevated late average.**

| **Group** | **Environment** | **Stable: n/N; % (95% CI)** | **Late elevated: n/N; % (95% CI)** |
| --- | --- | --- | --- |
| Previous hypertension diagnosis | Busy | 55/223; 24.7% (19.5 to 30.7) | 78/223; 35.0% (29.0 to 41.4) |
| No previous hypertension diagnosis | Busy | 71/220; 32.3% (26.4 to 38.7) | 30/220; 13.6% (9.7 to 18.8) |
| Previous hypertension diagnosis | Quiet | 64/223; 28.7% (23.2 to 35.0) | 73/223; 32.7% (26.9 to 39.1) |
| No previous hypertension diagnosis | Quiet | 66/220; 30.0% (24.3 to 36.4) | 21/220; 9.5% (6.3 to 14.2) |
| Stability requires each consecutive SBP change across 21,24,27,30 minutes to be strictly below 5 mmHg. It does not measure stability at 15 minutes. Elevated late average uses the 140/90 mmHg threshold applied to separate mean SBP/DBP values. Pointwise Wilson intervals; no diagnosis is made from this study alone. | | | |

**Table S10b. Temporal-window averaging sensitivity.**

| **Group** | **Environment** | **Averaged scheduled times (min)** | **Disagreement: n/N; % (95% CI)** |
| --- | --- | --- | --- |
| Previous hypertension diagnosis | Busy | 9,12 | 25/223; 11.2% (7.7 to 16.0) |
| No previous hypertension diagnosis | Busy | 9,12 | 16/220; 7.3% (4.5 to 11.5) |
| Previous hypertension diagnosis | Quiet | 9,12 | 28/223; 12.6% (8.8 to 17.5) |
| No previous hypertension diagnosis | Quiet | 9,12 | 9/220; 4.1% (2.2 to 7.6) |
| Previous hypertension diagnosis | Busy | 12,15 | 19/223; 8.5% (5.5 to 12.9) |
| No previous hypertension diagnosis | Busy | 12,15 | 10/220; 4.5% (2.5 to 8.2) |
| Previous hypertension diagnosis | Quiet | 12,15 | 21/223; 9.4% (6.2 to 14.0) |
| No previous hypertension diagnosis | Quiet | 12,15 | 10/220; 4.5% (2.5 to 8.2) |
| Previous hypertension diagnosis | Busy | 9,12,15 | 16/223; 7.2% (4.5 to 11.3) |
| No previous hypertension diagnosis | Busy | 9,12,15 | 13/220; 5.9% (3.5 to 9.8) |
| Previous hypertension diagnosis | Quiet | 9,12,15 | 24/223; 10.8% (7.3 to 15.5) |
| No previous hypertension diagnosis | Quiet | 9,12,15 | 9/220; 4.1% (2.2 to 7.6) |
| These are averages across different scheduled time points, not contemporaneous repeat readings at one time. The standard late average is retained. Pointwise Wilson intervals. Averaging does not necessarily reduce disagreement in every subgroup. | | | |

**Table S10c. Leave-one-out late-reference sensitivity.**

| **Group** | **Environment** | **Index time (min)** | **Disagreement: n/N; % (95% CI)** |
| --- | --- | --- | --- |
| No previous hypertension diagnosis | Quiet | 24 | 10/220; 4.5% (2.5 to 8.2) |
| Previous hypertension diagnosis | Quiet | 24 | 22/223; 9.9% (6.6 to 14.5) |
| No previous hypertension diagnosis | Busy | 24 | 15/220; 6.8% (4.2 to 10.9) |
| Previous hypertension diagnosis | Busy | 24 | 30/223; 13.5% (9.6 to 18.6) |
| No previous hypertension diagnosis | Quiet | 27 | 14/220; 6.4% (3.8 to 10.4) |
| Previous hypertension diagnosis | Quiet | 27 | 19/223; 8.5% (5.5 to 12.9) |
| No previous hypertension diagnosis | Busy | 27 | 16/220; 7.3% (4.5 to 11.5) |
| Previous hypertension diagnosis | Busy | 27 | 26/223; 11.7% (8.1 to 16.5) |
| No previous hypertension diagnosis | Quiet | 30 | 11/220; 5.0% (2.8 to 8.7) |
| Previous hypertension diagnosis | Quiet | 30 | 23/223; 10.3% (7.0 to 15.0) |
| No previous hypertension diagnosis | Busy | 30 | 16/220; 7.3% (4.5 to 11.5) |
| Previous hypertension diagnosis | Busy | 30 | 29/223; 13.0% (9.2 to 18.1) |
| At each late index time, the index is removed from the reference and classification is compared with the mean of the other two late readings. This removes self-inclusion but does not create an independent physiological resting baseline. Pointwise Wilson intervals. | | | |

**Table S11. Marginal fixed-effect tests in reporting models.**

| **Outcome** | **Term** | **Numerator df** | **Denominator df** | **F** | **Nominal p** |
| --- | --- | --- | --- | --- | --- |
| SBP | time_f | 10 | 8820 | 437.356 | <0.001 |
| SBP | environment | 1 | 440 | 64.280 | <0.001 |
| SBP | group | 1 | 435 | 4.574 | 0.033 |
| SBP | age_s1 | 1 | 435 | 32.413 | <0.001 |
| SBP | age_s2 | 1 | 435 | 22.197 | <0.001 |
| SBP | age_s3 | 1 | 435 | 6.352 | 0.012 |
| SBP | sex | 1 | 435 | 7.688 | 0.006 |
| SBP | BMI_c | 1 | 435 | 1.176 | 0.279 |
| SBP | period | 1 | 440 | 0.094 | 0.760 |
| SBP | sequence | 1 | 435 | 3.411 | 0.065 |
| SBP | time_f:environment | 10 | 8820 | 1.756 | 0.063 |
| SBP | time_f:group | 10 | 8820 | 5.280 | <0.001 |
| SBP | environment:group | 1 | 440 | 5.331 | 0.021 |
| SBP | time_f:environment:group | 10 | 8820 | 2.277 | 0.012 |
| DBP | time_f | 10 | 8820 | 148.447 | <0.001 |
| DBP | environment | 1 | 440 | 17.797 | <0.001 |
| DBP | group | 1 | 435 | 9.678 | 0.002 |
| DBP | age_s1 | 1 | 435 | 1.947 | 0.164 |
| DBP | age_s2 | 1 | 435 | 5.406 | 0.021 |
| DBP | age_s3 | 1 | 435 | 6.285 | 0.013 |
| DBP | sex | 1 | 435 | 1.817 | 0.178 |
| DBP | BMI_c | 1 | 435 | 9.575 | 0.002 |
| DBP | period | 1 | 440 | 1.955 | 0.163 |
| DBP | sequence | 1 | 435 | 5.234 | 0.023 |
| DBP | time_f:environment | 10 | 8820 | 1.668 | 0.082 |
| DBP | time_f:group | 10 | 8820 | 1.826 | 0.051 |
| DBP | environment:group | 1 | 440 | 4.300 | 0.039 |
| DBP | time_f:environment:group | 10 | 8820 | 1.349 | 0.198 |
| PR | time_f | 10 | 8820 | 23.574 | <0.001 |
| PR | environment | 1 | 440 | 81.834 | <0.001 |
| PR | group | 1 | 435 | 2.040 | 0.154 |
| PR | age_s1 | 1 | 435 | 5.226 | 0.023 |
| PR | age_s2 | 1 | 435 | 2.639 | 0.105 |
| PR | age_s3 | 1 | 435 | 0.038 | 0.845 |
| PR | sex | 1 | 435 | 8.867 | 0.003 |
| PR | BMI_c | 1 | 435 | 4.703 | 0.031 |
| PR | period | 1 | 440 | 0.235 | 0.628 |
| PR | sequence | 1 | 435 | 0.255 | 0.614 |
| PR | time_f:environment | 10 | 8820 | 1.061 | 0.389 |
| PR | time_f:group | 10 | 8820 | 12.526 | <0.001 |
| PR | environment:group | 1 | 440 | 6.477 | 0.011 |
| PR | time_f:environment:group | 10 | 8820 | 0.724 | 0.703 |
| PP | time_f | 10 | 8820 | 185.006 | <0.001 |
| PP | environment | 1 | 440 | 38.604 | <0.001 |
| PP | group | 1 | 435 | 0.369 | 0.544 |
| PP | age_s1 | 1 | 435 | 47.704 | <0.001 |
| PP | age_s2 | 1 | 435 | 22.104 | <0.001 |
| PP | age_s3 | 1 | 435 | 32.790 | <0.001 |
| PP | sex | 1 | 435 | 9.256 | 0.002 |
| PP | BMI_c | 1 | 435 | 1.784 | 0.182 |
| PP | period | 1 | 440 | 0.350 | 0.554 |
| PP | sequence | 1 | 435 | 0.564 | 0.453 |
| PP | time_f:environment | 10 | 8820 | 1.222 | 0.271 |
| PP | time_f:group | 10 | 8820 | 8.023 | <0.001 |
| PP | environment:group | 1 | 440 | 1.080 | 0.299 |
| PP | time_f:environment:group | 10 | 8820 | 1.439 | 0.156 |
| These tests retain the original nlme denominator degrees of freedom. Covariates and lower-order terms must be interpreted in the context of all interactions. p-values are nominal, and no coefficient-specific age-spline term is a stand-alone biological age effect. | | | | | |

**Table S12. Within-session residual lag correlations.**

| **Outcome** | **Lag (min)** | **Residual pairs** | **Correlation** |
| --- | --- | --- | --- |
| SBP | 3 | 8860 | -0.198 |
| SBP | 6 | 7974 | -0.039 |
| SBP | 9 | 7088 | -0.066 |
| SBP | 12 | 6202 | -0.067 |
| SBP | 15 | 5316 | -0.081 |
| SBP | 18 | 4430 | -0.078 |
| SBP | 21 | 3544 | -0.059 |
| SBP | 24 | 2658 | -0.049 |
| SBP | 27 | 1772 | -0.039 |
| SBP | 30 | 886 | -0.093 |
| DBP | 3 | 8860 | -0.149 |
| DBP | 6 | 7974 | -0.074 |
| DBP | 9 | 7088 | -0.093 |
| DBP | 12 | 6202 | -0.091 |
| DBP | 15 | 5316 | -0.073 |
| DBP | 18 | 4430 | -0.077 |
| DBP | 21 | 3544 | -0.064 |
| DBP | 24 | 2658 | -0.069 |
| DBP | 27 | 1772 | -0.034 |
| DBP | 30 | 886 | -0.007 |
| PR | 3 | 8860 | -0.156 |
| PR | 6 | 7974 | -0.048 |
| PR | 9 | 7088 | -0.080 |
| PR | 12 | 6202 | -0.067 |
| PR | 15 | 5316 | -0.083 |
| PR | 18 | 4430 | -0.081 |
| PR | 21 | 3544 | -0.108 |
| PR | 24 | 2658 | -0.048 |
| PR | 27 | 1772 | -0.092 |
| PR | 30 | 886 | 0.021 |
| PP | 3 | 8860 | -0.153 |
| PP | 6 | 7974 | -0.050 |
| PP | 9 | 7088 | -0.059 |
| PP | 12 | 6202 | -0.067 |
| PP | 15 | 5316 | -0.075 |
| PP | 18 | 4430 | -0.093 |
| PP | 21 | 3544 | -0.058 |
| PP | 24 | 2658 | -0.060 |
| PP | 27 | 1772 | -0.075 |
| PP | 30 | 886 | -0.125 |
| Descriptive pooled Pearson correlations using normalized conditional residuals at exact lags within participant/environment sessions; lag zero omitted. No cross-session pairs, significance thresholds or automatic model-adequacy claims. | | | |

**Table S13. Laboratory availability and reporting status.**

| **Marker** | **Group** | **N** | **Available** | **Quantified** | **Left** | **Right** | **Unresolved** | **Missing** |
| --- | --- | --- | --- | --- | --- | --- | --- | --- |
| Serum creatinine (micromol/L) | Previous hypertension diagnosis | 223 | 220 | 220 | 0 | 0 | 0 | 3 |
| Serum creatinine (micromol/L) | No previous hypertension diagnosis | 220 | 210 | 210 | 0 | 0 | 0 | 10 |
| Urine creatinine (mmol/L) | Previous hypertension diagnosis | 223 | 210 | 210 | 0 | 0 | 0 | 13 |
| Urine creatinine (mmol/L) | No previous hypertension diagnosis | 220 | 206 | 206 | 0 | 0 | 0 | 14 |
| Urine sodium (mmol/L) | Previous hypertension diagnosis | 223 | 210 | 193 | 17 | 0 | 0 | 13 |
| Urine sodium (mmol/L) | No previous hypertension diagnosis | 220 | 206 | 199 | 7 | 0 | 0 | 14 |
| Urine potassium (mmol/L) | Previous hypertension diagnosis | 223 | 210 | 210 | 0 | 0 | 0 | 13 |
| Urine potassium (mmol/L) | No previous hypertension diagnosis | 220 | 206 | 206 | 0 | 0 | 0 | 14 |
| Urine albumin (mg/L) | Previous hypertension diagnosis | 223 | 210 | 210 | 0 | 0 | 0 | 13 |
| Urine albumin (mg/L) | No previous hypertension diagnosis | 220 | 206 | 206 | 0 | 0 | 0 | 14 |
| Urine albumin/creatinine (mg/g) | Previous hypertension diagnosis | 223 | 210 | 210 | 0 | 0 | 0 | 13 |
| Urine albumin/creatinine (mg/g) | No previous hypertension diagnosis | 220 | 206 | 206 | 0 | 0 | 0 | 14 |
| Urine EGF (pg/mL) | Previous hypertension diagnosis | 223 | 219 | 199 | 0 | 17 | 3 | 4 |
| Urine EGF (pg/mL) | No previous hypertension diagnosis | 220 | 209 | 158 | 0 | 47 | 4 | 11 |
| Urine sodium/potassium (mmol/mmol) | Previous hypertension diagnosis | 223 | 210 | 193 | 17 | 0 | 0 | 13 |
| Urine sodium/potassium (mmol/mmol) | No previous hypertension diagnosis | 220 | 206 | 199 | 7 | 0 | 0 | 14 |
| Urine EGF/creatinine (ng/mmol) | Previous hypertension diagnosis | 223 | 209 | 190 | 0 | 16 | 3 | 14 |
| Urine EGF/creatinine (ng/mmol) | No previous hypertension diagnosis | 220 | 204 | 155 | 0 | 45 | 4 | 16 |
| Numbers of participants; missing tests and unresolved calibration entries are separate from censored results. EGF bounds are conditional on the kit range. | | | | | | | | |

**Table S14. Laboratory concentrations and ratio summaries.**

| **Marker** | **Group** | **N** | **Median [Q1; Q3]** |
| --- | --- | --- | --- |
| Serum creatinine (micromol/L) | Previous hypertension diagnosis | 220 | 76.0 [62.0; 97.0] |
| Serum creatinine (micromol/L) | No previous hypertension diagnosis | 210 | 68.0 [57.0; 81.0] |
| Urine creatinine (mmol/L) | Previous hypertension diagnosis | 210 | 4.71 [2.41; 8.23] |
| Urine creatinine (mmol/L) | No previous hypertension diagnosis | 206 | 5.51 [2.87; 8.88] |
| Urine sodium (mmol/L) | Previous hypertension diagnosis | 210 | 59.0 [34.0; 89.0] |
| Urine sodium (mmol/L) | No previous hypertension diagnosis | 206 | 72.0 [47.0; 104.0] |
| Urine potassium (mmol/L) | Previous hypertension diagnosis | 210 | 38.0 [22.0; 60.0] |
| Urine potassium (mmol/L) | No previous hypertension diagnosis | 206 | 36.0 [21.0; 60.0] |
| Urine albumin (mg/L) | Previous hypertension diagnosis | 210 | 4.5 [4.0; 9.3] |
| Urine albumin (mg/L) | No previous hypertension diagnosis | 206 | 4.7 [4.0; 6.8] |
| Urine albumin/creatinine (mg/g) | Previous hypertension diagnosis | 210 | 11.3 [6.1; 24.0] |
| Urine albumin/creatinine (mg/g) | No previous hypertension diagnosis | 206 | 9.0 [5.3; 20.9] |
| Urine EGF (pg/mL) | Previous hypertension diagnosis | 216 | 2203 [1221; 3780] |
| Urine EGF (pg/mL) | No previous hypertension diagnosis | 205 | 2997 [1755; 6867] |
| Urine sodium/potassium (mmol/mmol) | Previous hypertension diagnosis | 210 | [1.56, 1.67] [[0.88, 1.04]; [2.55, 2.56]] |
| Urine sodium/potassium (mmol/mmol) | No previous hypertension diagnosis | 206 | [2.04, 2.14] [[1.33, 1.38]; [2.88, 2.95]] |
| Urine EGF/creatinine (ng/mmol) | Previous hypertension diagnosis | 206 | [509, 512] [203; [1049, 1312]] |
| Urine EGF/creatinine (ng/mmol) | No previous hypertension diagnosis | 200 | [710, 840] [278; [1377, 4788]] |
| Empirical inverse-CDF quartiles (type 1). Inequalities or bracketed bounds indicate incompletely identified sample quantiles, not confidence intervals. Censored observations contribute bounds. Unresolved EGF values are excluded only from these numerical summaries. | | | |

**Table S15. Adjusted laboratory group comparisons.**

| **Marker** | **N** | **Ratio (95% CI)** | **Nominal p** | **Routine BH q (7)** | **Conditional EGF q (2)** |
| --- | --- | --- | --- | --- | --- |
| Serum creatinine | 430 | 1.01 (0.93 to 1.09) | 0.891 | 1.000 |  |
| Urine sodium | 416 | 0.94 (0.78 to 1.13) | 0.494 | 0.865 |  |
| Urine potassium | 416 | 1.18 (0.95 to 1.46) | 0.141 | 0.492 |  |
| Urine EGF (conditional) | 421 | 1.03 (0.80 to 1.32) | 0.834 |  | 0.975 |
| Urine EGF/creatinine (conditional) | 406 | 1.00 (0.78 to 1.30) | 0.975 |  | 0.975 |
| Urine sodium/potassium | 416 | 0.87 (0.75 to 1.02) | 0.091 | 0.492 |  |
| Urine albumin/creatinine | 416 | 1.14 (0.87 to 1.50) | 0.334 | 0.779 |  |
| Previous diagnosis divided by no previous diagnosis. Censored-lognormal models adjust for age spline, sex and BMI; Sodium/potassium and EGF/creatinine models also include log potassium and log urine creatinine, respectively; uACR models do not add urine creatinine. Routine-marker BH adjustment retains seven planned comparisons. EGF is conditional sensitivity, adjusted separately across its two group tests. Intervals are pointwise robust 95% intervals. | | | | | |

**Table S16. Laboratory associations with signed SBP difference at 12 and 15 minutes.**

| **Marker** | **Minute** | **N** | **Estimate (95% CI)** | **Nominal p** | **Routine BH q (14)** | **Conditional EGF q (4)** |
| --- | --- | --- | --- | --- | --- | --- |
| Serum creatinine | 12 | 430 | -0.90 (-2.40 to 0.59) | 0.237 | 0.758 |  |
| Serum creatinine | 15 | 430 | 0.48 (-0.68 to 1.64) | 0.421 | 0.758 |  |
| Urine sodium | 12 | 416 | -0.27 (-0.84 to 0.30) | 0.348 | 0.758 |  |
| Urine sodium | 15 | 416 | -0.21 (-0.73 to 0.31) | 0.433 | 0.758 |  |
| Urine potassium | 12 | 416 | -0.24 (-0.66 to 0.18) | 0.266 | 0.758 |  |
| Urine potassium | 15 | 416 | 0.03 (-0.34 to 0.40) | 0.867 | 1.000 |  |
| Urine EGF (conditional) | 12 | 421 | 0.00 (-0.39 to 0.39) | 0.982 |  | 0.992 |
| Urine EGF (conditional) | 15 | 421 | -0.14 (-0.50 to 0.22) | 0.447 |  | 0.920 |
| Urine EGF/creatinine (conditional) | 12 | 406 | 0.00 (-0.39 to 0.40) | 0.992 |  | 0.992 |
| Urine EGF/creatinine (conditional) | 15 | 406 | -0.14 (-0.51 to 0.23) | 0.460 |  | 0.920 |
| Urine sodium/potassium | 12 | 416 | -0.15 (-0.85 to 0.54) | 0.663 | 1.000 |  |
| Urine sodium/potassium | 15 | 416 | -0.31 (-0.97 to 0.35) | 0.361 | 0.758 |  |
| Urine albumin/creatinine | 12 | 416 | 0.16 (-0.17 to 0.49) | 0.351 | 0.758 |  |
| Urine albumin/creatinine | 15 | 416 | 0.24 (-0.02 to 0.51) | 0.075 | 0.758 |  |
| Outcome: time-point SBP minus the same-participant, same-environment average at 24,27,30 minutes. Estimates are mmHg per doubling, pooled across environments. The joint log-marker/bivariate-BP model integrates over censored predictor values, with participant-score sandwich intervals. Adjusted for age spline, sex, BMI, diagnostic group, environment, environment-by-group, period and sequence; Sodium/potassium and EGF/creatinine models also include log potassium and log urine creatinine, respectively; uACR models do not add urine creatinine. Routine BH q-values retain 14 planned marker-time comparisons. EGF tests are conditional and adjusted separately across four tests. This is not a prediction-validation study. | | | | | | |

**Table S17a. Quantified-only BP sensitivity analysis.**

| **Marker** | **Minute** | **N** | **Estimate (95% CI)** | **Nominal p** |
| --- | --- | --- | --- | --- |
| Serum creatinine | 12 | 430 | -0.92 (-2.43 to 0.59) | 0.232 |
| Serum creatinine | 15 | 430 | 0.48 (-0.69 to 1.65) | 0.422 |
| Urine sodium | 12 | 392 | -0.03 (-0.59 to 0.53) | 0.914 |
| Urine sodium | 15 | 392 | 0.14 (-0.45 to 0.72) | 0.645 |
| Urine potassium | 12 | 416 | -0.24 (-0.67 to 0.19) | 0.270 |
| Urine potassium | 15 | 416 | 0.03 (-0.34 to 0.40) | 0.867 |
| Urine EGF (conditional) | 12 | 357 | 0.24 (-0.31 to 0.78) | 0.392 |
| Urine EGF (conditional) | 15 | 357 | 0.06 (-0.42 to 0.53) | 0.816 |
| Urine EGF/creatinine (conditional) | 12 | 345 | 0.25 (-0.31 to 0.81) | 0.378 |
| Urine EGF/creatinine (conditional) | 15 | 345 | 0.03 (-0.45 to 0.51) | 0.912 |
| Urine sodium/potassium | 12 | 392 | 0.12 (-0.54 to 0.77) | 0.727 |
| Urine sodium/potassium | 15 | 392 | 0.16 (-0.53 to 0.85) | 0.652 |
| Urine albumin/creatinine | 12 | 416 | 0.15 (-0.18 to 0.49) | 0.365 |
| Urine albumin/creatinine | 15 | 416 | 0.24 (-0.03 to 0.51) | 0.078 |
| Quantified-only participant-clustered OLS; not a substitute for the full censored-data analysis. Estimates describe a linear association within the quantified subset. No participant is removed from the primary BP analysis. | | | | |

**Table S17b. Additional serum-creatinine adjustment in urinary-marker BP models.**

| **Marker** | **Minute** | **N** | **Estimate (95% CI)** | **Nominal p** |
| --- | --- | --- | --- | --- |
| Urine sodium | 12 | 412 | -0.29 (-0.84 to 0.27) | 0.317 |
| Urine sodium | 15 | 412 | -0.14 (-0.69 to 0.40) | 0.609 |
| Urine potassium | 12 | 412 | -0.26 (-0.67 to 0.16) | 0.227 |
| Urine potassium | 15 | 412 | 0.07 (-0.30 to 0.43) | 0.726 |
| Urine EGF (conditional) | 12 | 414 | -0.06 (-0.45 to 0.32) | 0.743 |
| Urine EGF (conditional) | 15 | 414 | -0.10 (-0.46 to 0.27) | 0.612 |
| Urine EGF/creatinine (conditional) | 12 | 402 | -0.07 (-0.45 to 0.31) | 0.722 |
| Urine EGF/creatinine (conditional) | 15 | 402 | -0.12 (-0.50 to 0.25) | 0.523 |
| Urine sodium/potassium | 12 | 412 | -0.16 (-0.85 to 0.53) | 0.647 |
| Urine sodium/potassium | 15 | 412 | -0.24 (-0.93 to 0.44) | 0.483 |
| Urine albumin/creatinine | 12 | 412 | 0.16 (-0.16 to 0.48) | 0.336 |
| Urine albumin/creatinine | 15 | 412 | 0.23 (-0.04 to 0.49) | 0.091 |
| Adds log serum creatinine; available cases may change. This is not adjustment for measured GFR or medication. Serum creatinine is not adjusted for itself. | | | | |

**Table S17c. EGF sensitivity applying both supplied kit limits.**

| **Marker** | **Minute** | **N** | **Left** | **Right** | **Estimate (95% CI)** | **Nominal p** |
| --- | --- | --- | --- | --- | --- | --- |
| Urine EGF (conditional) | 12 | 428 | 2 | 69 | 0.05 (-0.30 to 0.41) | 0.764 |
| Urine EGF (conditional) | 15 | 428 | 2 | 69 | -0.06 (-0.40 to 0.28) | 0.720 |
| Urine EGF/creatinine (conditional) | 12 | 413 | 2 | 66 | 0.05 (-0.30 to 0.41) | 0.766 |
| Urine EGF/creatinine (conditional) | 15 | 413 | 2 | 66 | -0.06 (-0.41 to 0.29) | 0.735 |
| All numerical EGF results above 8000 are treated as right-censored and those below 125 as left-censored. This is an explicit sensitivity assumption, not source-record confirmation of the seven additional entries. Undiluted-urine assay performance remains unestablished. | | | | | | |

**Table S17d. Laboratory group comparisons with additional serum-creatinine adjustment.**

| **Marker** | **N** | **Ratio (95% CI)** | **Nominal p** | **Routine BH q (7)** | **Conditional EGF q (2)** |
| --- | --- | --- | --- | --- | --- |
| Urine sodium | 412 | 0.97 (0.80 to 1.17) | 0.747 | 1.000 |  |
| Urine potassium | 412 | 1.20 (0.97 to 1.50) | 0.101 | 0.624 |  |
| Urine EGF (conditional) | 414 | 1.04 (0.80 to 1.35) | 0.780 |  | 0.976 |
| Urine EGF/creatinine (conditional) | 402 | 1.00 (0.77 to 1.31) | 0.976 |  | 0.976 |
| Urine sodium/potassium | 412 | 0.90 (0.76 to 1.05) | 0.178 | 0.624 |  |
| Urine albumin/creatinine | 412 | 1.13 (0.86 to 1.49) | 0.389 | 0.908 |  |
| Models add log serum creatinine; available samples may change. Sodium/potassium and EGF/creatinine models also include log potassium and log urine creatinine, respectively; uACR models do not add urine creatinine. Pointwise 95% intervals. This is not measured-GFR or medication adjustment. | | | | | |

**Table S17e. Conditional EGF group comparisons using both kit boundaries.**

| **Marker** | **N** | **Quantified** | **Below range** | **Above range** | **Ratio (95% CI)** | **Nominal p** | **Conditional BH q (2)** |
| --- | --- | --- | --- | --- | --- | --- | --- |
| Urine EGF (conditional) | 428 | 357 | 2 | 69 | 1.03 (0.80 to 1.33) | 0.828 | 0.967 |
| Urine EGF/creatinine (conditional) | 413 | 345 | 2 | 66 | 1.01 (0.77 to 1.31) | 0.967 | 0.967 |
| Applies 125/8000 pg/mL boundaries to all out-of-range entries as a sensitivity assumption. This does not validate undiluted urine testing. EGF/creatinine also conditions on urine creatinine. | | | | | | | |

**Table S18. Characteristics by laboratory availability.**

| **Marker** | **Availability** | **N** | **Previous diagnosis, n** | **Female, n** | **Mean age, years** | **Mean BMI, kg/m²** | **Mean signed SBP difference at 15 min, mmHg** |
| --- | --- | --- | --- | --- | --- | --- | --- |
| Serum creatinine | Available | 430 | 220 | 309 | 51.4 | 29.3 | 1.22 |
| Serum creatinine | Missing | 13 | 3 | 10 | 52.6 | 30.7 | -1.46 |
| Urine creatinine | Available | 416 | 210 | 302 | 51.3 | 29.4 | 1.18 |
| Urine creatinine | Missing | 27 | 13 | 17 | 52.4 | 28.6 | 0.44 |
| Urine sodium | Available | 416 | 210 | 302 | 51.3 | 29.4 | 1.18 |
| Urine sodium | Missing | 27 | 13 | 17 | 52.4 | 28.6 | 0.44 |
| Urine potassium | Available | 416 | 210 | 302 | 51.3 | 29.4 | 1.18 |
| Urine potassium | Missing | 27 | 13 | 17 | 52.4 | 28.6 | 0.44 |
| Urine albumin | Available | 416 | 210 | 302 | 51.3 | 29.4 | 1.18 |
| Urine albumin | Missing | 27 | 13 | 17 | 52.4 | 28.6 | 0.44 |
| Urine albumin/creatinine | Available | 416 | 210 | 302 | 51.3 | 29.4 | 1.18 |
| Urine albumin/creatinine | Missing | 27 | 13 | 17 | 52.4 | 28.6 | 0.44 |
| Urine EGF (conditional) | Available | 428 | 219 | 307 | 51.4 | 29.3 | 1.10 |
| Urine EGF (conditional) | Missing | 15 | 4 | 12 | 51.9 | 29.1 | 2.20 |
| Urine sodium/potassium | Available | 416 | 210 | 302 | 51.3 | 29.4 | 1.18 |
| Urine sodium/potassium | Missing | 27 | 13 | 17 | 52.4 | 28.6 | 0.44 |
| Urine EGF/creatinine (conditional) | Available | 413 | 209 | 300 | 51.3 | 29.4 | 1.16 |
| Urine EGF/creatinine (conditional) | Missing | 30 | 14 | 19 | 52.5 | 28.3 | 0.86 |
| Descriptive available-versus-missing comparisons do not establish the missing-data mechanism. | | | | | | | |

**Table S19. Questionnaire response distributions.**

| **Characteristic** | **Response** | **Hypertension** | **No previous diagnosis** |
| --- | --- | --- | --- |
| Ever had blood glucose checked | Missing | 0/223 (0.0) | 0/220 (0.0) |
| Ever had blood glucose checked | No | 21/223 (9.4) | 128/220 (58.2) |
| Ever had blood glucose checked | Uncertain | 1/223 (0.4) | 2/220 (0.9) |
| Ever had blood glucose checked | Yes | 201/223 (90.1) | 90/220 (40.9) |
| Previous diabetes diagnosis | Missing | 1/223 (0.4) | 1/220 (0.5) |
| Previous diabetes diagnosis | No | 156/223 (70.0) | 90/220 (40.9) |
| Previous diabetes diagnosis | Uncertain | 22/223 (9.9) | 127/220 (57.7) |
| Previous diabetes diagnosis | Yes | 44/223 (19.7) | 2/220 (0.9) |
| Reported alcohol use | Missing | 0/223 (0.0) | 0/220 (0.0) |
| Reported alcohol use | No | 173/223 (77.6) | 168/220 (76.4) |
| Reported alcohol use | Yes | 50/223 (22.4) | 52/220 (23.6) |
| Reported any current medication | Missing | 0/223 (0.0) | 0/220 (0.0) |
| Reported any current medication | No | 1/223 (0.4) | 218/220 (99.1) |
| Reported any current medication | Yes | 222/223 (99.6) | 2/220 (0.9) |
| Reported BP-related lifestyle modification | Missing | 0/223 (0.0) | 0/220 (0.0) |
| Reported BP-related lifestyle modification | No | 1/223 (0.4) | 219/220 (99.5) |
| Reported BP-related lifestyle modification | Uncertain | 0/223 (0.0) | 0/220 (0.0) |
| Reported BP-related lifestyle modification | Yes | 222/223 (99.6) | 1/220 (0.5) |
| Reported BP medication | Missing | 0/223 (0.0) | 0/220 (0.0) |
| Reported BP medication | No | 1/223 (0.4) | 220/220 (100.0) |
| Reported BP medication | Uncertain | 0/223 (0.0) | 0/220 (0.0) |
| Reported BP medication | Yes | 222/223 (99.6) | 0/220 (0.0) |
| Reported diabetes medication | Missing | 0/223 (0.0) | 0/220 (0.0) |
| Reported diabetes medication | No | 185/223 (83.0) | 219/220 (99.5) |
| Reported diabetes medication | Yes | 38/223 (17.0) | 1/220 (0.5) |
| Cells show n/N (%). Uncertain and missing diabetes responses are not No. Previous glucose testing is a history response, not a glucose measurement. | | | |

**Table S20a. Medication classes among participants with a previous hypertension diagnosis.**

| **Name interpretation** | **Class** | **Identified** | **Not identified** | **Unknown** |
| --- | --- | --- | --- | --- |
| Literal/confirmed | Beta-blocker | 76 | 106 | 41 |
| Literal/confirmed | Angiotensin-receptor blocker | 159 | 35 | 29 |
| Literal/confirmed | ACE inhibitor | 1 | 171 | 51 |
| Literal/confirmed | Calcium-channel blocker | 83 | 94 | 46 |
| Literal/confirmed | Thiazide/thiazide-like diuretic | 96 | 90 | 37 |
| Literal/confirmed | Mineralocorticoid-receptor antagonist | 46 | 129 | 48 |
| Literal/confirmed | Loop diuretic | 13 | 162 | 48 |
| Literal/confirmed | Direct vasodilator | 3 | 170 | 50 |
| Literal/confirmed | Glucose-lowering medicine | 29 | 148 | 46 |
| Literal/confirmed | Statin | 8 | 164 | 51 |
| Literal/confirmed | Antiplatelet | 3 | 170 | 50 |
| Literal/confirmed | Anticoagulant | 0 | 172 | 51 |
| Literal/confirmed | Nitrate | 1 | 171 | 51 |
| Proposed spellings | Beta-blocker | 79 | 120 | 24 |
| Proposed spellings | Angiotensin-receptor blocker | 163 | 37 | 23 |
| Proposed spellings | ACE inhibitor | 1 | 196 | 26 |
| Proposed spellings | Calcium-channel blocker | 86 | 111 | 26 |
| Proposed spellings | Thiazide/thiazide-like diuretic | 97 | 100 | 26 |
| Proposed spellings | Mineralocorticoid-receptor antagonist | 53 | 144 | 26 |
| Proposed spellings | Loop diuretic | 13 | 184 | 26 |
| Proposed spellings | Direct vasodilator | 3 | 195 | 25 |
| Proposed spellings | Glucose-lowering medicine | 30 | 169 | 24 |
| Proposed spellings | Statin | 11 | 186 | 26 |
| Proposed spellings | Antiplatelet | 3 | 194 | 26 |
| Proposed spellings | Anticoagulant | 1 | 196 | 26 |
| Proposed spellings | Nitrate | 1 | 196 | 26 |
| Categories overlap. Literal/confirmed names and proposed-spelling assumptions are reported separately. Not identified does not establish non-use; incomplete lists remain unknown unless the class is identified. | | | | |

**Table S20b. Generic ingredients identified from the recorded lists.**

| **Generic ingredient** | **Overall** | **Hypertension** | **No previous diagnosis** |
| --- | --- | --- | --- |
| amlodipine | 79 | 79 | 0 |
| aspirin | 2 | 2 | 0 |
| atorvastatin | 3 | 3 | 0 |
| bendroflumethiazide | 2 | 2 | 0 |
| bisoprolol | 21 | 21 | 0 |
| budesonide | 1 | 0 | 1 |
| candesartan | 34 | 34 | 0 |
| carbidopa | 1 | 1 | 0 |
| carvedilol | 32 | 32 | 0 |
| clopidogrel | 1 | 1 | 0 |
| esomeprazole | 1 | 0 | 1 |
| furosemide | 12 | 12 | 0 |
| glibenclamide | 1 | 1 | 0 |
| glimepiride | 16 | 16 | 0 |
| hydralazine | 3 | 3 | 0 |
| hydrochlorothiazide | 93 | 93 | 0 |
| insulin | 2 | 2 | 0 |
| irbesartan | 24 | 24 | 0 |
| isosorbide dinitrate | 1 | 1 | 0 |
| levodopa | 1 | 1 | 0 |
| lisinopril | 1 | 1 | 0 |
| losartan | 71 | 71 | 0 |
| metformin | 23 | 23 | 0 |
| metolazone | 1 | 1 | 0 |
| metoprolol | 21 | 21 | 0 |
| montelukast | 1 | 0 | 1 |
| nifedipine | 2 | 2 | 0 |
| propranolol | 2 | 2 | 0 |
| rosuvastatin | 5 | 5 | 0 |
| s-amlodipine | 3 | 3 | 0 |
| sitagliptin | 6 | 5 | 1 |
| spironolactone | 46 | 46 | 0 |
| telmisartan | 30 | 30 | 0 |
| torsemide | 1 | 1 | 0 |
| Literal/confirmed mappings only. Combination products contribute each ingredient once per participant. No dose timing, adherence or frequency is inferred. | | | |

**Table S21. Matched-sample clinical sensitivity comparisons.**

| **Outcome / addition** | **Group** | **Time (min)** | **n** | **Reference (95% CI)** | **Adjusted (95% CI)** | **Adjusted Holm p** |
| --- | --- | --- | --- | --- | --- | --- |
| SBP / Alcohol | No previous diagnosis | 12 | 443 | 4.66 (3.21 to 6.10) | 4.66 (3.21 to 6.10) | <0.001 |
| SBP / Alcohol | Hypertension | 12 | 443 | 1.83 (0.40 to 3.27) | 1.83 (0.40 to 3.27) | 0.062 |
| SBP / Alcohol | No previous diagnosis | 15 | 443 | 4.07 (2.58 to 5.56) | 4.07 (2.58 to 5.56) | <0.001 |
| SBP / Alcohol | Hypertension | 15 | 443 | 2.87 (1.39 to 4.35) | 2.87 (1.39 to 4.35) | 0.002 |
| SBP / Diabetes + alcohol | No previous diagnosis | 12 | 292 | 5.50 (3.26 to 7.74) | 5.50 (3.26 to 7.74) | <0.001 |
| SBP / Diabetes + alcohol | Hypertension | 12 | 292 | 1.60 (0.08 to 3.12) | 1.60 (0.08 to 3.12) | 0.294 |
| SBP / Diabetes + alcohol | No previous diagnosis | 15 | 292 | 4.25 (1.95 to 6.55) | 4.25 (1.95 to 6.55) | 0.005 |
| SBP / Diabetes + alcohol | Hypertension | 15 | 292 | 2.68 (1.13 to 4.24) | 2.68 (1.13 to 4.24) | 0.011 |
| DBP / Alcohol | No previous diagnosis | 12 | 443 | 2.18 (1.16 to 3.20) | 2.18 (1.16 to 3.20) | <0.001 |
| DBP / Alcohol | Hypertension | 12 | 443 | 0.43 (-0.58 to 1.45) | 0.43 (-0.58 to 1.45) | 1.000 |
| DBP / Alcohol | No previous diagnosis | 15 | 443 | 1.37 (0.32 to 2.42) | 1.37 (0.32 to 2.42) | 0.158 |
| DBP / Alcohol | Hypertension | 15 | 443 | 1.01 (-0.03 to 2.06) | 1.01 (-0.03 to 2.06) | 0.671 |
| DBP / Diabetes + alcohol | No previous diagnosis | 12 | 292 | 2.23 (0.66 to 3.81) | 2.23 (0.66 to 3.81) | 0.108 |
| DBP / Diabetes + alcohol | Hypertension | 12 | 292 | 0.29 (-0.78 to 1.36) | 0.29 (-0.78 to 1.36) | 1.000 |
| DBP / Diabetes + alcohol | No previous diagnosis | 15 | 292 | 1.58 (-0.03 to 3.19) | 1.58 (-0.03 to 3.19) | 0.874 |
| DBP / Diabetes + alcohol | Hypertension | 15 | 292 | 0.77 (-0.32 to 1.87) | 0.77 (-0.32 to 1.87) | 1.000 |
| PR / Alcohol | No previous diagnosis | 12 | 443 | 2.17 (1.28 to 3.06) | 2.17 (1.28 to 3.06) | <0.001 |
| PR / Alcohol | Hypertension | 12 | 443 | 1.39 (0.51 to 2.28) | 1.39 (0.51 to 2.28) | 0.017 |
| PR / Alcohol | No previous diagnosis | 15 | 443 | 2.40 (1.49 to 3.31) | 2.40 (1.49 to 3.31) | <0.001 |
| PR / Alcohol | Hypertension | 15 | 443 | 1.34 (0.44 to 2.24) | 1.34 (0.44 to 2.24) | 0.026 |
| PR / Diabetes + alcohol | No previous diagnosis | 12 | 292 | 1.87 (0.56 to 3.18) | 1.87 (0.56 to 3.18) | 0.060 |
| PR / Diabetes + alcohol | Hypertension | 12 | 292 | 1.38 (0.49 to 2.27) | 1.38 (0.49 to 2.27) | 0.034 |
| PR / Diabetes + alcohol | No previous diagnosis | 15 | 292 | 2.14 (0.81 to 3.47) | 2.14 (0.81 to 3.47) | 0.026 |
| PR / Diabetes + alcohol | Hypertension | 15 | 292 | 1.31 (0.41 to 2.21) | 1.31 (0.41 to 2.21) | 0.055 |
| SBP / Medication; literal | Hypertension | 12 | 176 | 1.45 (-0.24 to 3.14) | 1.45 (-0.24 to 3.14) | 0.463 |
| SBP / Medication; literal | Hypertension | 15 | 176 | 2.50 (0.75 to 4.25) | 2.50 (0.76 to 4.24) | 0.047 |
| SBP / Medication; proposed | Hypertension | 12 | 197 | 1.72 (0.14 to 3.30) | 1.72 (0.14 to 3.30) | 0.201 |
| SBP / Medication; proposed | Hypertension | 15 | 197 | 2.87 (1.23 to 4.50) | 2.87 (1.23 to 4.50) | 0.006 |
| Busy minus quiet: mmHg for SBP/DBP; beats/min for pulse. Reference and adjusted models use the same participants within each comparison. Pointwise 95% intervals; Holm p-values retain the original within-model comparison families. | | | | | | |

**Table S22a. Pulse-rate contrasts by beta-blocker identification.**

| **Name interpretation** | **Time (min)** | **n** | **Pulse difference (95% CI)** | **Nominal p** | **Holm p** |
| --- | --- | --- | --- | --- | --- |
| Literal/confirmed | 0 | 182 | -4.80 (-8.85 to -0.74) | 0.021 | 0.101 |
| Literal/confirmed | 3 | 182 | -5.41 (-9.46 to -1.35) | 0.009 | 0.101 |
| Literal/confirmed | 6 | 182 | -5.14 (-9.19 to -1.09) | 0.013 | 0.101 |
| Literal/confirmed | 9 | 182 | -5.76 (-9.81 to -1.71) | 0.006 | 0.101 |
| Literal/confirmed | 12 | 182 | -5.88 (-9.93 to -1.82) | 0.005 | 0.095 |
| Literal/confirmed | 15 | 182 | -6.23 (-10.29 to -2.17) | 0.003 | 0.062 |
| Literal/confirmed | 18 | 182 | -5.73 (-9.79 to -1.68) | 0.006 | 0.101 |
| Literal/confirmed | 21 | 182 | -5.75 (-9.81 to -1.69) | 0.006 | 0.101 |
| Literal/confirmed | 24 | 182 | -5.71 (-9.77 to -1.65) | 0.006 | 0.101 |
| Literal/confirmed | 27 | 182 | -5.36 (-9.42 to -1.29) | 0.010 | 0.101 |
| Literal/confirmed | 30 | 182 | -5.58 (-9.65 to -1.51) | 0.007 | 0.101 |
| Proposed spellings | 0 | 199 | -5.34 (-9.19 to -1.50) | 0.007 | 0.043 |
| Proposed spellings | 3 | 199 | -5.99 (-9.84 to -2.15) | 0.002 | 0.043 |
| Proposed spellings | 6 | 199 | -5.61 (-9.45 to -1.76) | 0.004 | 0.043 |
| Proposed spellings | 9 | 199 | -6.00 (-9.85 to -2.15) | 0.002 | 0.043 |
| Proposed spellings | 12 | 199 | -6.11 (-9.96 to -2.26) | 0.002 | 0.040 |
| Proposed spellings | 15 | 199 | -6.37 (-10.22 to -2.53) | 0.001 | 0.028 |
| Proposed spellings | 18 | 199 | -5.95 (-9.80 to -2.10) | 0.003 | 0.043 |
| Proposed spellings | 21 | 199 | -5.98 (-9.83 to -2.13) | 0.003 | 0.043 |
| Proposed spellings | 24 | 199 | -5.91 (-9.77 to -2.06) | 0.003 | 0.043 |
| Proposed spellings | 27 | 199 | -5.50 (-9.35 to -1.64) | 0.005 | 0.043 |
| Proposed spellings | 30 | 199 | -5.71 (-9.57 to -1.85) | 0.004 | 0.043 |
| Identified minus not identified; beats/min. The model constrains this contrast to be common across environments, so it is displayed once. Original Holm p-values are preserved. This is an observational comparison; proposed spellings are conditional. | | | | | |

**Table S22b. Between-list-group differences in pulse-rate change from time zero.**

| **Name interpretation** | **Time (min)** | **Difference in change (95% CI)** | **Nominal p** | **Holm p** |
| --- | --- | --- | --- | --- |
| Literal/confirmed | 3 | -0.61 (-1.48 to 0.26) | 0.168 | 1.000 |
| Literal/confirmed | 6 | -0.35 (-1.39 to 0.70) | 0.516 | 1.000 |
| Literal/confirmed | 9 | -0.97 (-2.08 to 0.15) | 0.089 | 1.000 |
| Literal/confirmed | 12 | -1.08 (-2.23 to 0.06) | 0.064 | 1.000 |
| Literal/confirmed | 15 | -1.43 (-2.60 to -0.27) | 0.016 | 0.311 |
| Literal/confirmed | 18 | -0.94 (-2.11 to 0.24) | 0.117 | 1.000 |
| Literal/confirmed | 21 | -0.96 (-2.14 to 0.23) | 0.113 | 1.000 |
| Literal/confirmed | 24 | -0.92 (-2.11 to 0.27) | 0.131 | 1.000 |
| Literal/confirmed | 27 | -0.56 (-1.76 to 0.64) | 0.359 | 1.000 |
| Literal/confirmed | 30 | -0.78 (-1.99 to 0.43) | 0.205 | 1.000 |
| Proposed spellings | 3 | -0.65 (-1.47 to 0.18) | 0.124 | 1.000 |
| Proposed spellings | 6 | -0.26 (-1.26 to 0.73) | 0.602 | 1.000 |
| Proposed spellings | 9 | -0.66 (-1.72 to 0.41) | 0.226 | 1.000 |
| Proposed spellings | 12 | -0.77 (-1.87 to 0.33) | 0.169 | 1.000 |
| Proposed spellings | 15 | -1.03 (-2.15 to 0.08) | 0.069 | 1.000 |
| Proposed spellings | 18 | -0.61 (-1.73 to 0.51) | 0.288 | 1.000 |
| Proposed spellings | 21 | -0.64 (-1.77 to 0.49) | 0.269 | 1.000 |
| Proposed spellings | 24 | -0.57 (-1.71 to 0.57) | 0.326 | 1.000 |
| Proposed spellings | 27 | -0.16 (-1.31 to 0.99) | 0.790 | 1.000 |
| Proposed spellings | 30 | -0.37 (-1.53 to 0.79) | 0.533 | 1.000 |
| Derived contrasts of the absolute-pulse model, in beats/min, shown once because they are common across environments. Original multiplicity correction is retained. | | | | |

**Table S22c. Joint time-by-beta-blocker tests for pulse rate.**

| **Name interpretation** | **n** | **Wald chi-square** | **df** | **p** |
| --- | --- | --- | --- | --- |
| Literal/confirmed | 182 | 8.560 | 10 | 0.574 |
| Proposed spellings | 199 | 6.787 | 10 | 0.745 |
| Asymptotic Wald chi-square tests, not likelihood-ratio or common-denominator F-tests. Nonsignificance does not establish equivalent trajectories. | | | | |
